# Multiscale structure and function of the aortic valve apparatus

**DOI:** 10.1152/physrev.00038.2022

**Published:** 2023-09-21

**Authors:** Hussam El-Nashar, Malak Sabry, Yuan-Tsan Tseng, Nadine Francis, Najma Latif, Kim H. Parker, James E. Moore, Magdi H. Yacoub

**Affiliations:** ^1^Aswan Heart Research Centre, Magdi Yacoub Foundation, Cairo, Egypt; ^2^Department of Bioengineering, Imperial College London, London, United Kingdom; ^3^Department of Biomedical Engineering, King’s College London, London, United Kingdom; ^4^Heart Science Centre, Magdi Yacoub Institute, London, United Kingdom; ^5^National Heart and Lung Institute, Imperial College London, London, United Kingdom

**Keywords:** aortic root, aortic valve, biomechanics, molecular, developmental and cellular mechanisms, structure-function relationships

## Abstract

Whereas studying the aortic valve in isolation has facilitated the development of life-saving procedures and technologies, the dynamic interplay of the aortic valve and its surrounding structures is vital to preserving their function across the wide range of conditions encountered in an active lifestyle. Our view is that these structures should be viewed as an integrated functional unit, here referred to as the aortic valve apparatus (AVA). The coupling of the aortic valve and root, left ventricular outflow tract, and blood circulation is crucial for AVA’s functions: unidirectional flow out of the left ventricle, coronary perfusion, reservoir function, and support of left ventricular function. In this review, we explore the multiscale biological and physical phenomena that underlie the simultaneous fulfillment of these functions. A brief overview of the tools used to investigate the AVA, such as medical imaging modalities, experimental methods, and computational modeling, specifically fluid-structure interaction (FSI) simulations, is included. Some pathologies affecting the AVA are explored, and insights are provided on treatments and interventions that aim to maintain quality of life. The concepts explained in this article support the idea of AVA being an integrated functional unit and help identify unanswered research questions. Incorporating phenomena through the molecular, micro, meso, and whole tissue scales is crucial for understanding the sophisticated normal functions and diseases of the AVA.

## Graphical abstract

**Figure:**
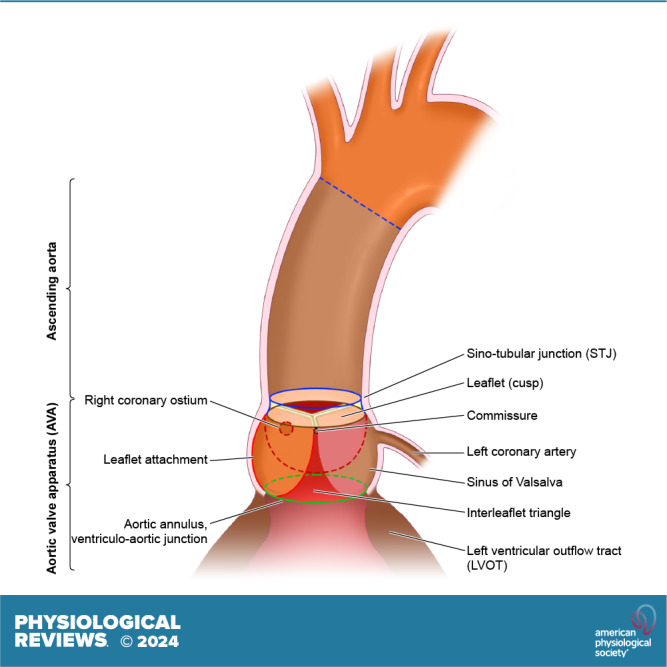

CLINICAL HIGHLIGHTS This review demonstrates the inherent elegance coupled with complexity of the aortic valve apparatus (AVA). Understanding this could have important clinical and translational implications in optimizing the management of patients with aortic valve disease to enhance longevity and quality of life. Understanding the structure and function of the AVA is essential in:

- Planning the preoperative procedure
- Matching the patient-specific pathologies to the surgical intervention
- Enhancing patient survival and quality of life.

## 1. INTRODUCTION: CONTEXT OF THE AORTIC VALVE APPARATUS

The aortic valve is an elegant structure responsible for performing sophisticated functions. Its function has been most widely recognized as maintaining tightly regulated, responsive, energy-efficient unidirectional blood flow out of the heart. However, more recently the aortic valve has been shown to anticipate hemodynamic events and to perform and contribute to additional functions: from coronary perfusion to contributing to the reservoir function required to maintain diastolic blood pressure and supporting left ventricular function under the widely varying loading conditions imposed by an active lifestyle. Furthermore, there has been strong evidence that the valve’s functions are also served by the complex surrounding tissues in subtle but important ways. The process of integrating back up to the macro level must therefore extend beyond the leaflets and annulus. Here, we present an overview of the macro and micro components of the parts of the aortic valve apparatus (AVA),^1^ which act in a coordinated fashion to perform the elegant, complex, and dynamic functions. In addition, we review the tools used to define and analyze these functions in the laboratory as well as in the clinic. We also review some of the ways that disruption of these interactions is involved in valvular pathologies and what interactions need to be retained in the surgical repair of the aortic valve.

## 2. STRUCTURE OF THE AVA COMPONENTS

**FIGURE 1.**
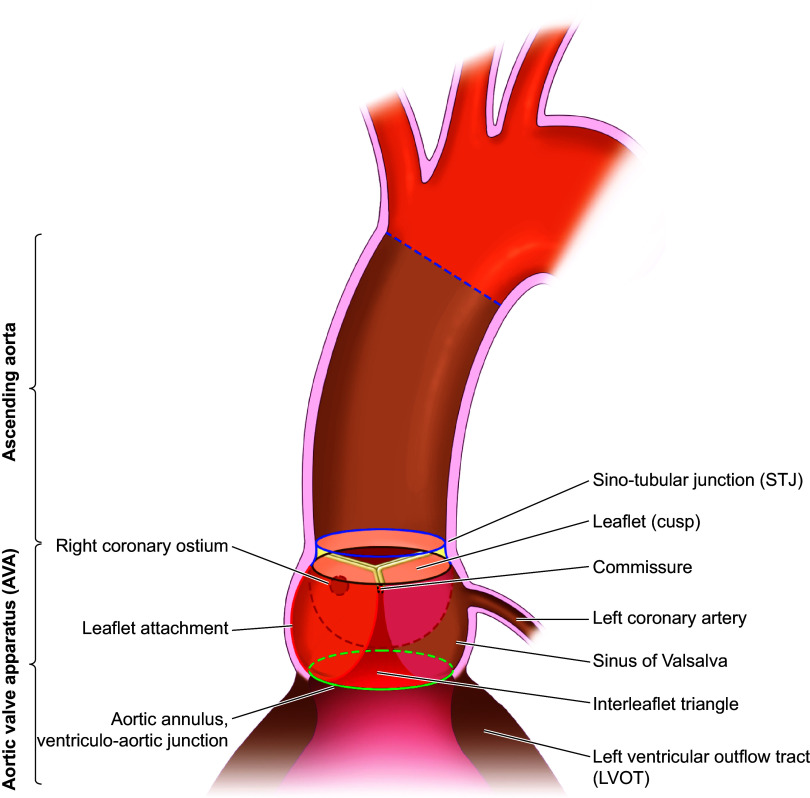
Proposed anatomy and nomenclature of the aortic valve apparatus (AVA), which includes all the tissue from the beginning of the left ventricle (LV) outflow tract (LVOT) in the LV to the sino-tubular junction (STJ) at the beginning of the ascending aorta. This includes the LVOT that is embedded in the LV myocardium, the 3 interleaflet triangles, the 3 sinuses of Valsalva, the left and right coronary ostia, the 3 leaflets, the 3 commissure points, and the STJ.

The macrostructure of the AVA includes all of the tissue from the junction of the left ventricle (LV) outflow tract (LVOT) to the start of the ascending aorta where the sinuses of Valsalva end. This point is frequently called the sino-tubular junction (STJ), although there is some disagreement about its anatomical definition, whether it is at the commissure level or further downstream where the bulging of the sinuses end and the more cylindrical ascending aorta starts (FIGURE 1). We choose the latter. Included in the AVA are the aortic valve leaflets, which are joined to the walls of the aorta and the fibrous body within the walls at the sinuses of Valsalva (sinuses), which are the three dilated portions of the aortic wall associated with the three aortic valve leaflets. We also note that the AVA encompasses a complex structure sometimes referred to as the “aortic root.” Although this term lacks a clear definition, we will define the aortic root as all the tissue from the start of the LVOT at the LV to the STJ, excluding the leaflets, encompassing the three sinuses of Valsalva and the three interleaflet triangles.

### 2.1. The Aortic Valve

#### 2.1.1. Geometric and functional characteristics.

**FIGURE 2.**
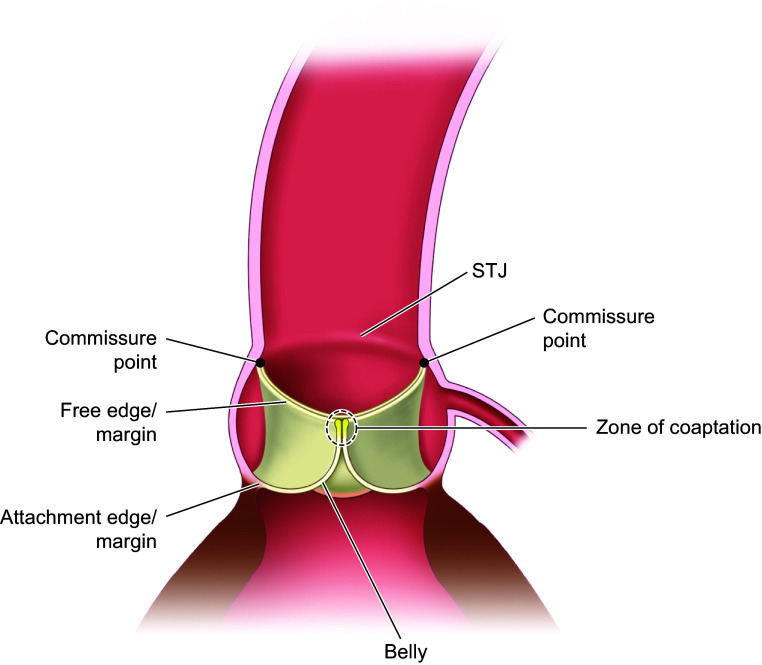
Leaflet definitions displayed through a cross-sectional cut through the middle of the aortic valve apparatus (AVA) during diastole.

Ideal aortic valve function involves opening fully during systole and closing completely during diastole, ensuring unidirectional blood flow, avoidance of damage to blood cells, and minimal or no regurgitation. Improper closing and opening are key indicators of a diseased valve. The shape of the AVA and its kinematics deeply affect these functions. The leaflets and sinuses are shaped in a way to facilitate proper function of the valve. The semilunar leaflet consists of a belly region and coaptation area, bounded by the free margin/edge and the fixed/attachment edge that attaches the leaflet to the sinus (FIGURE 2), where the leaflets’ bellies allow for a large luminal area during systole. During diastole, the shapes of the leaflets’ bellies, along with the three sinuses, engulf ∼14 mL of blood at arterial blood pressure (4) that pushes the leaflets together to coapt. The length of the leaflet’s free margin/edge is also longer than the straight-line distances between the commissures, with a ratio of ∼1.4 for the leaflet’s free edge length to the commissure distances (5, 6). This difference is related to the structural properties of the leaflets and is necessary to prevent stenotic opening while minimizing regurgitation when closed (5). The relatively flat region below the free margin/edge and above the belly of the leaflet provides the area required for coaptation, which prevents regurgitation during diastole (7). The coaptation zone is defined by the contact area of any two leaflets when the valve is fully closed, as shown in FIGURE 2. The curved geometry of the cusps has been shown to play a major role in reducing the stress distributions in the leaflets (8).

**FIGURE 3.**
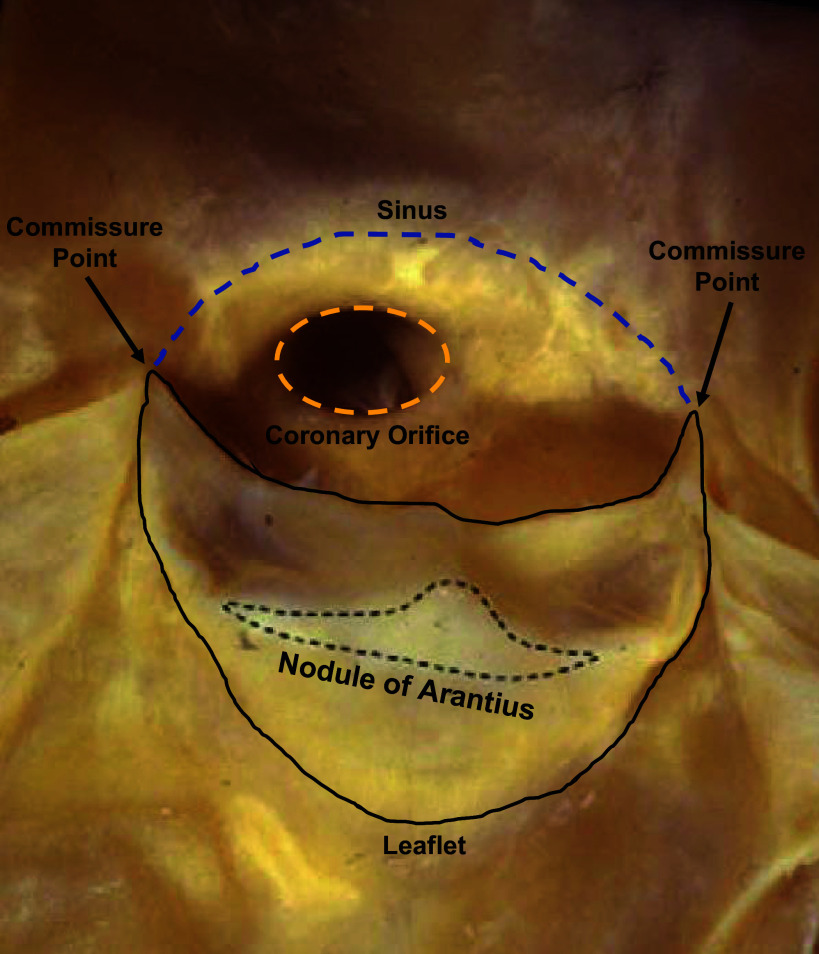
Nodule of Arantius (enclosed by the black dotted line) on the ventricular surface of the leaflet (enclosed by the black line). The leaflet height is smaller than the sinus height. The coronary ostium’s (enclosed by the yellow dotted line) position is superior to the leaflet and inferior to the sino-tubular junction (STJ). Adapted from Ref. 9, with permission from *European Journal of Echocardiography*.

At the bottom of the coaptation area where it meets the belly, on the ventricular side, is the nodule of Arantius (FIGURE 3), which is a thickened mass of elastic tissue (9). It has been shown that on the ventricular side there is a considerable amount of elastin (10) that stores energy during the systolic phase and helps the valve restore its original shape during the diastolic phase (11).

**FIGURE 4.**
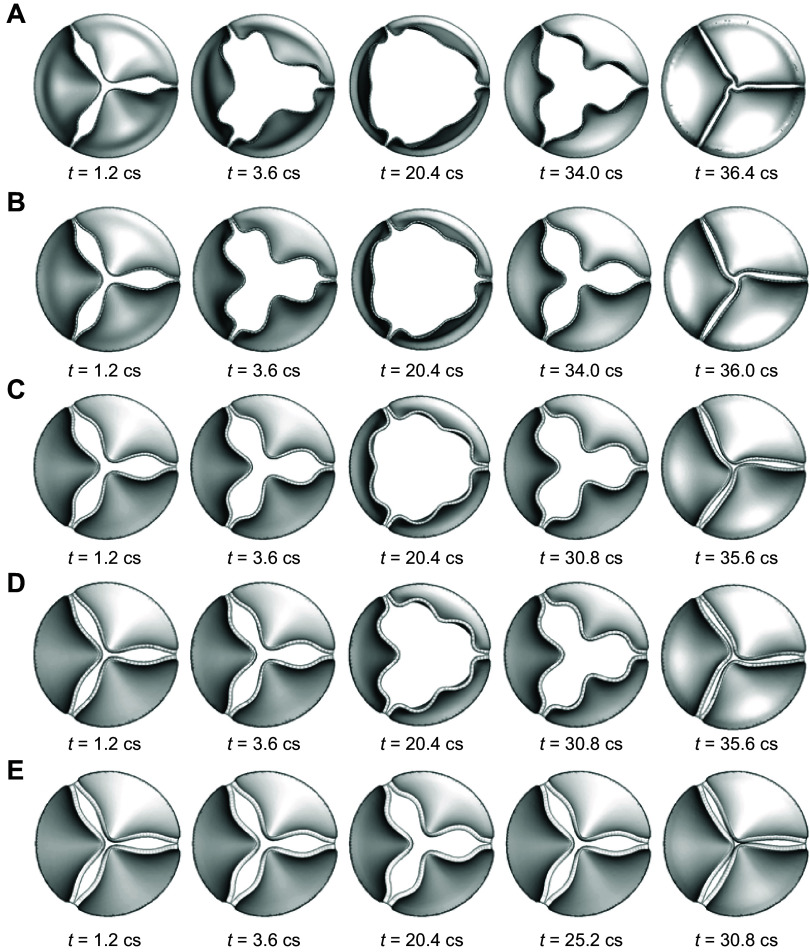
Three-dimensional (3-D) fluid-structure interaction (FSI) simulation results for aortic valve deformations during opening and closing for leaflet thickness 0.2 mm (*A*), 0.3 mm (*B*), 0.5 mm (*C*), 0.6 mm (*D*), and 0.8 mm (*E*). Adapted from Ref. 12, with permission from *Journal of Fluid Mechanics*.

The leaflets’ thickness also plays an important role in valve function. In general, the greater the overall or average thickness, the higher the structural and bending rigidity, which leads to higher leaflet stiffness and resistance to deformation. This rigidity depends on the geometry and material properties, which have a significant effect on the dynamic shape and extent of the valve opening. This means that a thickened leaflet will lead to smaller valve orifice areas, resulting in more obstruction to the flow, whereas a thinner leaflet will exhibit an exaggerated fluttering motion. Computational modeling of different thicknesses of the leaflets shows that the change in thickness could alter the instantaneous shape of the aortic orifice during systole and diastole (FIGURE 4) (12, 13). The distorted orifices change the flow stream, which in turn affects the transvalvular pressure drop and stress distributions in the leaflets. The rate of change in pressure produces varying effects on the receptor-mediated function of the endothelium analogous to structural changes in the vessel walls (14). Higher stresses can lead to calcification and thickening of the leaflets. It has been observed that the belly region and the leaflet attachment edge exhibit high stress concentrations that coincide with calcification initiation sites (15). Leaflets thickened by disease undergo higher shear stresses that could disrupt valve function and lead to valve failure. Additionally, shear stresses greater than ∼40 Pa (16) can lead to platelet adhesion or calcification and eventually valve failure (13, 17). These changes in leaflet biomechanics have been connected to endothelial dysfunction, which initiates a positive feedback loop toward calcification via activation of valvular interstitial cells toward an osteogenic phenotype (18–21).

Normal leaflets have a nonuniform thickness, slightly thicker toward the free edge and slightly thinner toward the fixed edge (5). An increase in diastolic pressure can accentuate the thickness difference as the belly of the leaflet stretches more (22). The role of the nonuniform thickness in function is not entirely clear. The spatial resolution of in vivo imaging modalities is insufficient to capture variations in leaflet thickness, leading to reliance on postmortem measurement to capture the correct variation in thickness along the leaflets. Computational analysis using fluid-structure interaction (FSI) simulations could also help identify potential roles of nonuniform leaflet thickness in valve kinematics and function through systematic variation of thickness geometry. This requires choosing appropriate output parameters, which depends on the context of the specific hypothesis or question being addressed.

**FIGURE 5.**
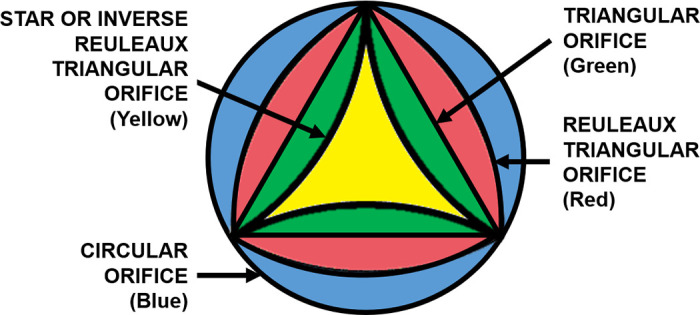
Schematic representation of the superimposed aortic valve orifices during systole. Adapted from Ref. 32, with permission per Open Access terms.

The opening shape of the valve is a three-lobed propellor configuration formed by the free edges of the leaflets, as shown in FIGURE 4 (12). This has been consistently presented in experimental studies of aortic valves (23–25) as well as FSI simulations (26–30). This three-lobed configuration is produced by the high stiffness of the inward fold at the middle of the free edge, which the leaflets have to snap through to overcome this shape and extend outward to create an opposite curvature (12). This configuration starts to form while opening and closing, during early and late systole, respectively. This three-arm star shape becomes approximately triangular before and after midsystole. At midsystole, the cusps form a Reuleaux triangle, which is a triangular shape with curved sides, where the length of any straight line through the center and intersecting the triangle is constant for all angles (31) (FIGURE 5). The normal open valve has a triangular orifice during ∼50% of the systolic phase. The triangular orifice arises because the leaflets are in tension from the expanded aortic diameter (33).

The fully open shapes of the valve are indicative of whether it is healthy or diseased. A healthy aortic valve’s orifice increases in size and becomes more circular to accommodate a greater flow during peak systole (34). Diseases such as aortic root aneurysm and Marfan syndrome increase the stretching out of leaflets, limiting the fully open shape to a triangular orifice with straight sides (35). The effects of the Reuleaux triangular shape versus the straight-sided triangular orifices on the function of the AVA and blood flow have not been fully explored, which could be interesting.

**FIGURE 6.**
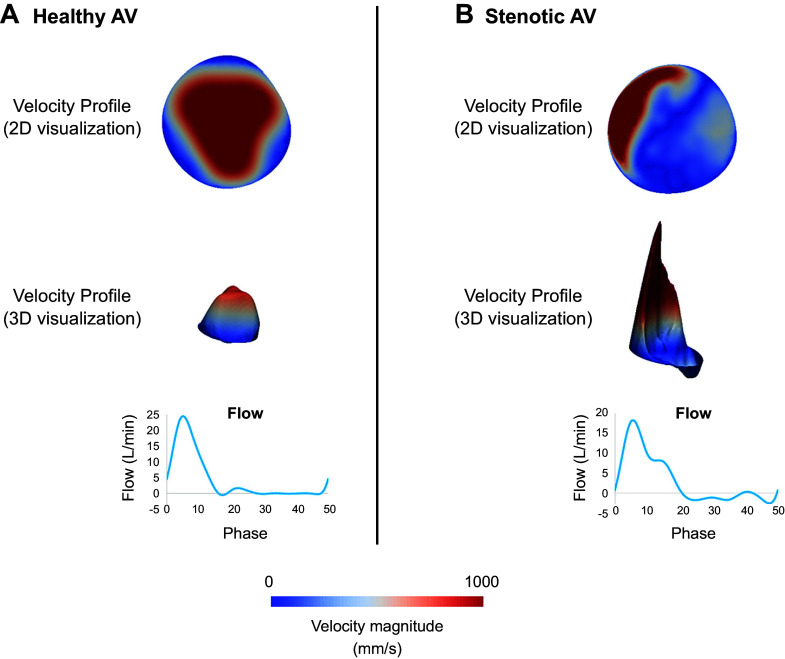
Two (2-D)- and three (3-D)-dimensional velocity profiles for healthy aortic valve (AV) (*A*) and stenotic AV (*B*). Adapted from Ref. 38, with permission from *Journal of Biomechanical Engineering*.

Some ventricular, valvular, and aortic diseases can change the aortic orifice area, shape, and size, consequently affecting valve function and downstream flow patterns. Aortic orifice area is usually defined using the geometric orifice area (GOA) and effective orifice area (EOA). GOA is the anatomical measurement of the area between the leaflets by planimetry, whereas EOA is defined as the minimal cross-sectional area of the aortic jet downstream of the valve, which is highly dependent on the valve inflow shape and is usually smaller than the GOA (36). Furthermore, the orifice shape depends on several factors such as blood flow and compliance of the root. A reduction in left ventricular contractility can cause the valve to open more slowly and have a smaller EOA (37). A change in the shape or size of the valve’s orifice area, due to stenosis for instance, can lead to disturbed and chaotic flow (FIGURE 6). This results in a highly turbulent flow, which could damage red blood cells and the endothelial cells lining the ascending aorta’s wall (39). The interpretation of the severity of aortic stenosis depends on the understanding of the interaction between cardiac output and the structure of the aortic valve as exemplified by paradoxical low-flow, low-gradient severe aortic stenosis (40, 41).

#### 2.1.2. Material characteristics.

Healthy leaflets have a complex structure consisting of three layers: the fibrosa, the spongiosa, and the ventricularis. The fibrosa and the ventricularis are composed primarily of collagen and elastin, respectively, whereas the spongiosa is mainly composed of proteoglycans and glycosaminoglycans (GAGs). The collagen fibers in the fibrosa are the stress-bearing components required for maintaining the tissue’s structural integrity. The elastin fibers in the ventricularis are the elastic component responsible for recoiling and restoring the stretched tissue and wavy and crimpled state of collagen fibers. The proteoglycans and GAGs in the spongiosa are responsible for conferring flexibility, dampening vibrations, and resisting delamination (42). Furthermore, proteoglycans and GAGs in the spongiosa have been shown to influence tissue extensibility and stress decay in atrioventricular valves, which could also have an implication in the aortic valve (43, 44).

**FIGURE 7.**
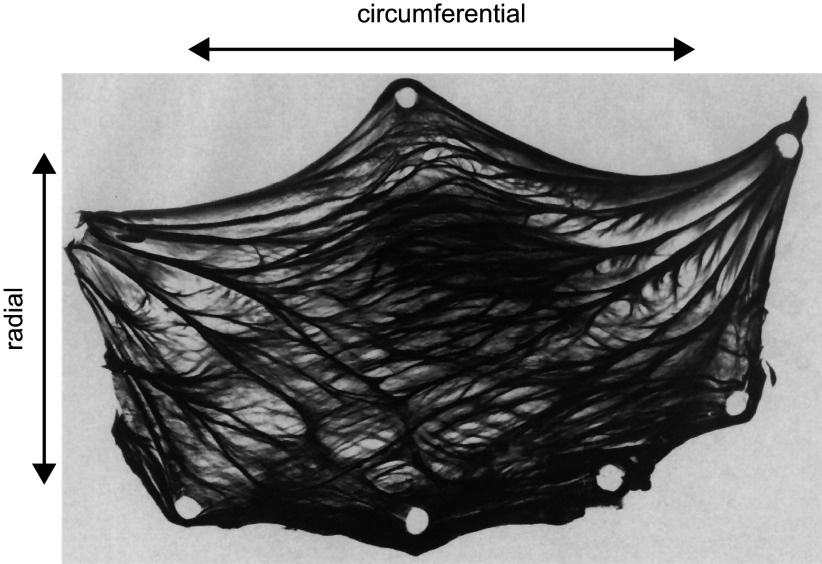
Collagen fibers of the aortic leaflet. The top of the leaflet sample is the free edge, and the semicircular bottom edge is the line of attachment to the walls of the sinus. Adapted from Ref. 46, with permission from the *American Physiological Society*.

This trilayered structure of the valves ensures high tensile strength required for withstanding the high transvalvular pressures during diastole and low flexural stiffness needed for normal opening and closing of the valve during systole (45). By small-angle light scattering (SALS), the collagen fiber directions of bovine aortic valve leaflets were observed to be coarsely aligned in the circumferential direction. The fibers start at one commissure end and generally course along the circumferential direction of the leaflet until they reach the other commissure point. The free edge is composed of a band of fibers oriented along the upper leaflet boundary (FIGURE 7) (47). The orientation of these fibers indicates that the material is anisotropic, or at the least transversely isotropic (48). This means that the leaflets can stretch more easily in the radial direction (across the fiber direction) than in the circumferential direction (along the fiber direction) (48, 49). The maximal principal stresses in the leaflets are also approximately aligned with the coarse collagen fiber direction. This anisotropic property of the leaflets is important to ensure proper coaptation.

The specific mechanical properties of the valve and root’s tissue tend to even out the stress and strain distributions within the tissue, ideally suited for diastolic valve function. This also appears to be relevant to mechanotransduction and cell mechanics (50). The cellular components of the valves, which include the valvular endothelial and interstitial cells, are key in the homeostasis, dynamism, and pathophysiology of the valve and are responsible for the superior performance of living valves compared with other valve substitutes (51–53).

To characterize the mechanical properties of the tissue, and since cardiac valve tissue is considered incompressible, planar biaxial testing is usually performed (54). Equibiaxial testing of porcine aortic leaflets has shown that the ratio of radial to circumferential stretch is 6.0 ± 1.1 (49). It has also been reported that aortic valves endure in vivo strains of 30.8% and 10.1%, with corresponding physiological strain rates of ∼12.4 ± 1.6 s^−1^ and 4.4 ± 0.8 s^−1^, in the principal radial and circumferential directions, respectively. This corresponds to an approximate 3-to-1 ratio of radial to circumferential strain and strain rate (55). These findings indicate that that there is a strong effect of the underlying fiber architecture on the two-dimensional strain distribution, i.e., the local tissue is much more compliant perpendicular to the preferred fiber orientation (radial direction) than along the fiber orientation (circumferential direction) (27).

Changes in the material properties or orientation of the fibers affect the leaflet kinematics and hence will influence the function and flow patterns. Several diseases alter the material properties of the valve, including calcification and rheumatic heart disease. These diseases introduce changes in the material structure of the leaflet by producing calcified nodules and causing fibrosis. These alterations result in regurgitation and aortic insufficiency due to increased stiffness (39, 56–61). Moreover, altered or increased stresses and strains induced in the valve are known to cause deterioration and calcification in the area of leaflet flexion (62) and tear in the leaflets near the commissures (63).

### 2.2. The Aortic Root

#### 2.2.1. Root geometry.

The aortic valve acts in cooperation with the aortic root complex. The aortic valve is composed of the three leaflets, whereas here we define the aortic root complex as composed of all the tissue from the start of LVOT in the ventricle to the STJ, excluding the leaflets: the LVOT, the three interleaflet fibrous triangles that are the proximal root portion between the ventricle at the annulus and the aortic valve, the three sinus bulges, part of the ascending aorta, and the two coronary ostia (9, 64). Together, the aortic root complex and the aortic valve form the AVA. Downstream of the STJ is the rest of the aorta, including the aortic arch and its branches. These distal features have at most some minor effects on flow in the sinus region but contribute greatly to the aorta’s ability to distribute half of the left ventricular stroke volume during diastole (65).

#### 2.2.2. Ascending aorta and aortic root complex tissue.

**FIGURE 8.**
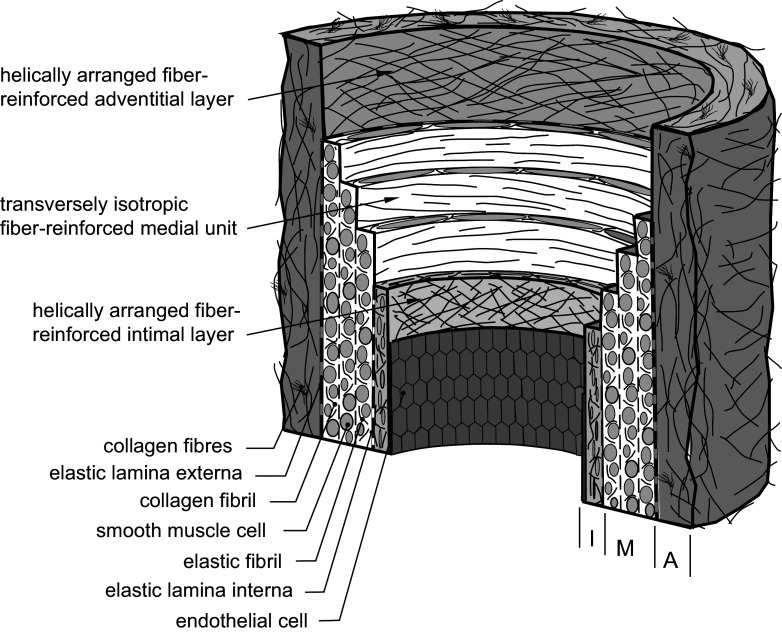
The microstructure of a healthy aorta, composed of the intima (I), the media (M), and the adventitia (A). The relative thicknesses of the three layers are only indicative and vary with axial location. Adapted from Ref. 66, with permission from *Journal of The Royal Society Interface*.

The structure of the aorta plays an important role in the function of the aortic valve. Its tissue is strong and highly elastic to withstand high blood pressures and aids in the aortic valve’s kinematics. The ascending aortic root tissue consists of three layers: the intima, media, and adventitia (FIGURE 8). In the adventitial and intimal layers, there are two families of collagen fibers that are helically intertwined (FIGURE 8), unlike the circumferential alignment in the aortic leaflets described previously (66). In the media layer, the structured circumferential alignment of the elastin and collagen bundles (FIGURE 8), along with the tension contributed by the contraction of the smooth muscle cells to resist loads (67), gives the aorta its ability to withstand high loads in the circumferential direction. Similarly to the aortic leaflets, the ascending aorta tissue demonstrates nonlinear hyperelastic behavior in both circumferential and longitudinal directions (68). It also contains a proportionately high elastin and low collagen content, making it a highly elastic vessel (9), with smooth muscle cells being the predominant cell type, crucial for maintaining wall integrity (69, 70). Compared with other sections of the aorta, the aortic sinuses exhibit a high degree of compliance at low pressures but then stiffen to a greater degree at higher pressures. (68). The mechanical properties of aortic leaflets, sinuses, and ascending aorta have been studied in depth (48, 71), whereas those of the interleaflet triangles have been scarcely investigated. An in vitro study of bioprosthetic valves has shown that changes in aortic root stiffness affect valve function (72). Aging produces progressive changes in the physical properties of the aorta (73–75). Genetic abnormalities, such as Marfan syndrome and tetralogy of Fallot, cause a reduced elasticity in the ascending aorta, which increases the chance of aortic root dilation and type A dissection (76, 77).

The elastic and compliant nature of the aorta has an important role in the aortic root’s kinematics, which in turn supports the aortic valve function. The distal portion of the aortic root expands because of pressure changes during systole, allowing the leaflets to retract and open. On the other end, the proximal part of the root expands during ventricle filling (diastole), allowing leaflets to open during early systole. It then contracts throughout systole, leading to a decrease in the distance the leaflets need to travel to coapt, causing the valve to close faster and the leaflets to coapt better (64). The proximal root expands and contracts with the LV, as they are conjoined; however, there is an ongoing discussion regarding the mechanics underlying aortic root expansion and contraction (33, 78, 79). Can the root’s dilation and contraction be explained solely by passive fluid dynamics and LV motion, or is there also an active process (80) or further physiological and pathological process that influences this motion?

#### 2.2.3. Vasa vasorum.

The adventitia and the outer two-thirds of the media of the vessel walls are vascularized by small vessels (vasa vasorum). These play an essential role in health and disease (81–83) by supplying nutrients as well as, importantly, cellular immune interaction (84). The influence of stripping the adventitia during surgical operations for congenital heart disease, such as the arterial switch operation, the Ross operation, or the Nikaidoh repair of the transposition of the great arteries (TGA), could have important influence on valve function in the longer term (85–87).

### 2.3. Aortic Valve Apparatus Innervation

**FIGURE 9.**
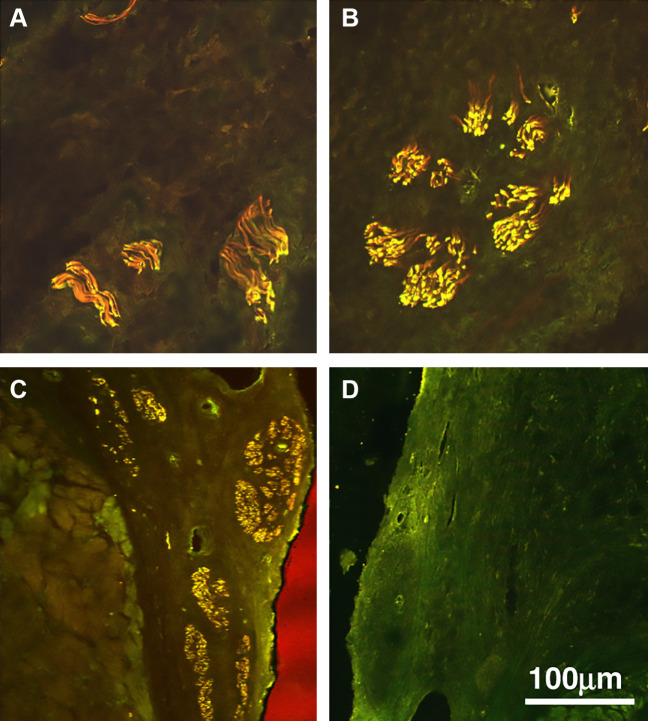
Confocal images of the nerves in the sino-tubular junction (STJ) (*A*), sinus (*B*), annulus (*C*), and control (*D*). Red is neurofilament protein (NF), green is choline acetyl transferase (ChAT), and yellow is colocalization of both NF and ChAT. Adapted from Ref. 88, with permission from *Journal of Molecular and Cellular Cardiology*.

**FIGURE 10.**
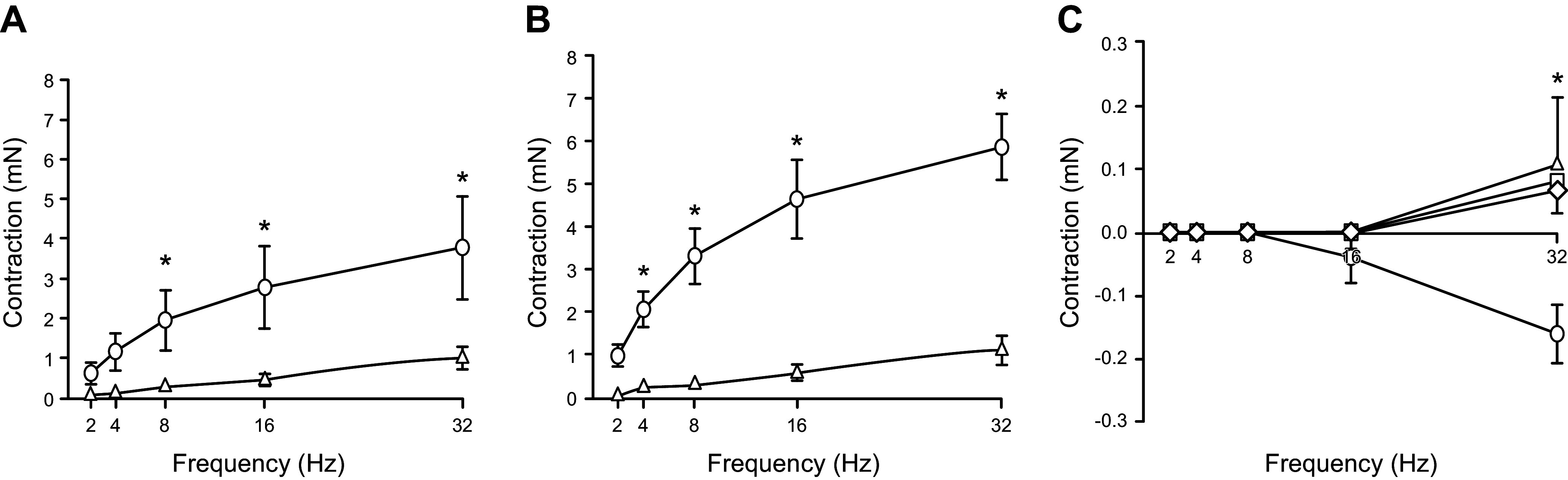
Electrical field stimulation frequency response curves for sino-tubular junction (STJ) tissue (*A*), sinus tissue (*B*), and leaflet tissue (*C*). Adapted from Ref. 88, with permission from *Journal of Molecular and Cellular Cardiology*.

All the aortic leaflets, the aortic annulus, sinuses, and STJ are densely innervated (FIGURE 9). These nerves appear to have important regulatory functions in the contractile function of the aortic leaflets and sinuses, which is mainly dilatory in the leaflet and constrictor in the sinuses (FIGURE 10) (88–91).

## 3. INTEGRATION: DYNAMISM AND CROSS TALK

### 3.1. Dynamism

**FIGURE 11.**
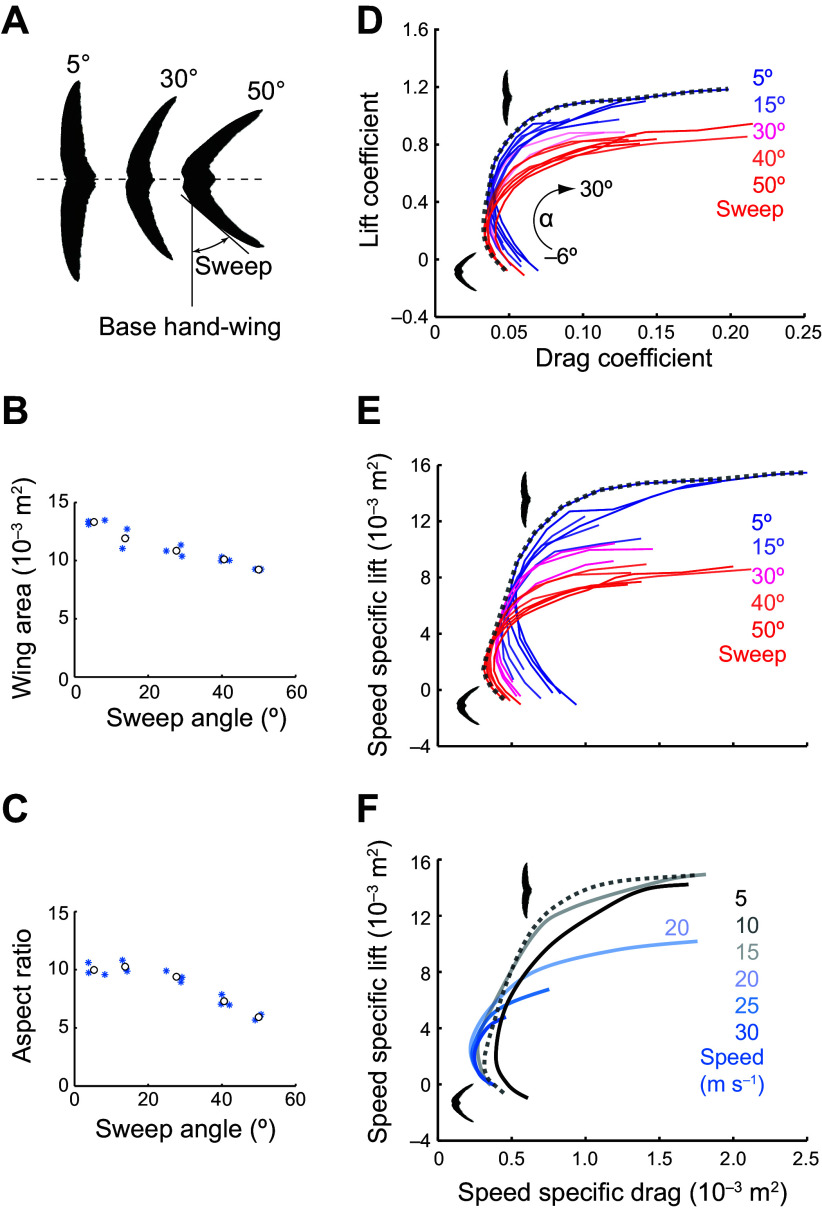
The relationship between size and shape of the wings of a swift and the speed of lift during flight. *A*–*C*: adjusting sweep angle (*A*) alters wing area (*B*) and aspect ratio (*C*). *D*: lift and drag coefficients for different sweep angles at a glide speed of 10 m/s, where α = angle of attack. *E* and *F*: effect of sweep angle (*E*) and glide speed (*F*) on lift and drag. Adapted from Ref. 96, with permission from *Nature*.

The components of the AVA exhibit complex modes of interaction that vary throughout the cardiac cycle. These interactions have been referred to as “dynamism” and “cross talk” (92). This dynamic behavior influences the pattern of instantaneous movements of the aortic leaflets and valve function (92). For example, the aortic root expansion before ejection (93) and its movement during systole lead to the speculation that the dynamics of the aortic root are responsible for facilitating opening of the valve (94). The movement of the interleaflet triangles could also be an important factor for optimum valve opening and closure. The movement of the root toward the LV against the blood applies forces on the leaflets that initiate valve opening. The STJ also expands during systole, separating the tops of the commissures that in turn move the free borders of the leaflets, causing the leaflets to open smoothly and form the straight-sided and Reuleaux triangular orifices. Similar mechanisms cause partial closure of the valve in late systole (92–94). Aortic root movement parameters have also been shown to correlate significantly with left ventricular function parameters (95). Changes in the size and shape of the different components of the AVA, including the leaflets, can play an important part in the precision of movement in a manner somewhat analogous to those observed in the changes in the size and shape of the wings of a swift during flight (FIGURE 11) (44, 96).

Dimensional changes in the aortic root expansion and contraction occur simultaneously with the opening and closing of the valve. It follows that the longevity of the natural aortic leaflets could be enhanced by a dynamic aortic root and, conversely, that the deterioration of stented prosthetic aortic leaflets could be attributed to the fact that they are attached to relatively stiff stents (33, 92).

Another facet of dynamism is torsion of the aortic root between the aortic base and the commissures. It is speculated that this aortic root twist reduces shear strains induced by the LV contractions, since it moves in the same direction. It is also suggested that this mechanism may minimize opening and closing shear stresses on the leaflets (97). Others have hypothesized that this twisting motion is an energy-storing mechanism. During diastole, energy is accumulated from the torsion of the root. Then, during systole, the aortic root twists back and releases this stored elastic energy back into the blood flow (98). Furthermore, the deformation of the aortic root is nonuniformly affected by left ventricular volume, pressure, and contractility, which vary during isovolumic contraction, ejection, isovolumic relaxation, and diastole (99).

These three-dimensional (3-D) aortic root deformations are also speculated to minimize stresses on the leaflets by optimizing leaflet loading conditions and reducing turbulence-related energy losses through the valve (99). In the absence of the compliance coming from the sinuses, leaflets have less space when traveling from their closed to their full opening position. This creates folds in the leaflets’ free edges, thus increasing stresses and accelerating the rate of failure (100). The radius of curvature of the sinuses will also decrease between systole and diastole to accommodate the differing levels of stress (101, 102).

**FIGURE 12.**
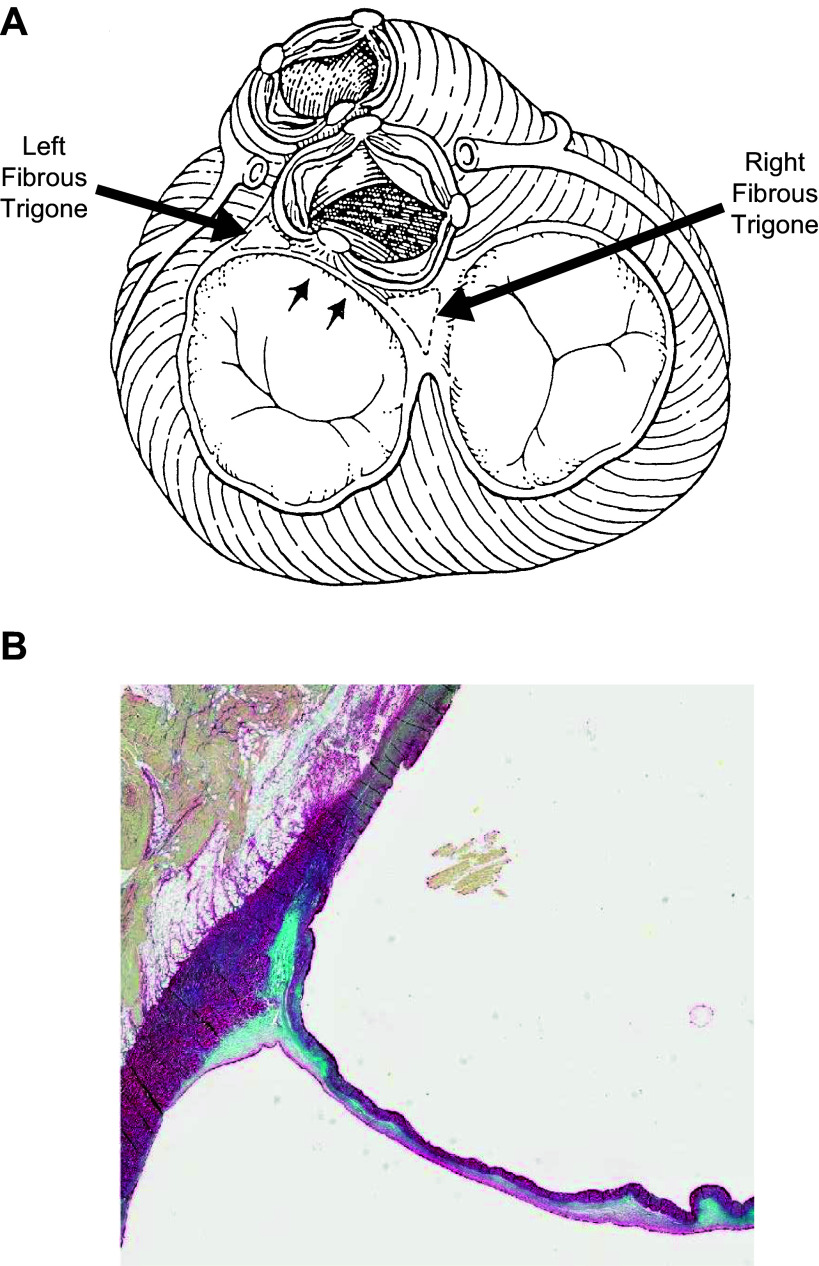
*A*: the hinge mechanism located between the left and right fibrous trigones (location indicated by the two small arrows). Adapted from Ref. 92, with permission from *Annals of Thoracic Surgery*. *B*: stained section through the noncoronary sinus and leaflet showing the attachment of the hinge to the right fibrous trigone. The blue stain shows the glycosaminoglycans (GAGs), whereas the red stain shows the collagen content. Adapted from Ref. 103, with permission per Open Access terms.

Furthermore, the right and left fibrous trigones in between the anterior and posterior left ventricular outflow tract (LVOT) walls act as hinge points (FIGURE 12*A*) that enable displacement of the root’s posterior wall in systole (101). This hinge mechanism has a major role in optimizing the shape and dynamism of the aortic valve orifice. The different layers in the hinge form a trilamellar structure that provides it with malleability, elasticity, and strength to aid the valve to function efficiently. This leads to the “trilamellar sliding” hypothesis introduced recently of the hinge achieving its multiple functions by the sliding of its three different layers over each other, with the aid of hydrophilic glycosaminoglycans (GAGs) in its core (FIGURE 12*B*) (103).

### 3.2. Cross talk

In addition to structural interactions, cellular communication with extracellular matrix (ECM) also contributes to AVA active dynamism. This interaction is referred to as cross talk (92). The cellular components involved in the valvular cross talk are the valvular endothelial cells (VECs) and valvular interstitial cells (VICs). The VECs form a monolayer of cells lining the valves that separate the blood components from the underlying VICs and ECM to provide antithrombogenic, anti-inflammatory properties and regulatory function. It has been shown there are differences in VECs and vascular endothelial cells (ECs) in both transcriptional profile and proliferation rate (104). This indicates that VECs have specialized functions compared with vascular ECs.

**FIGURE 13.**
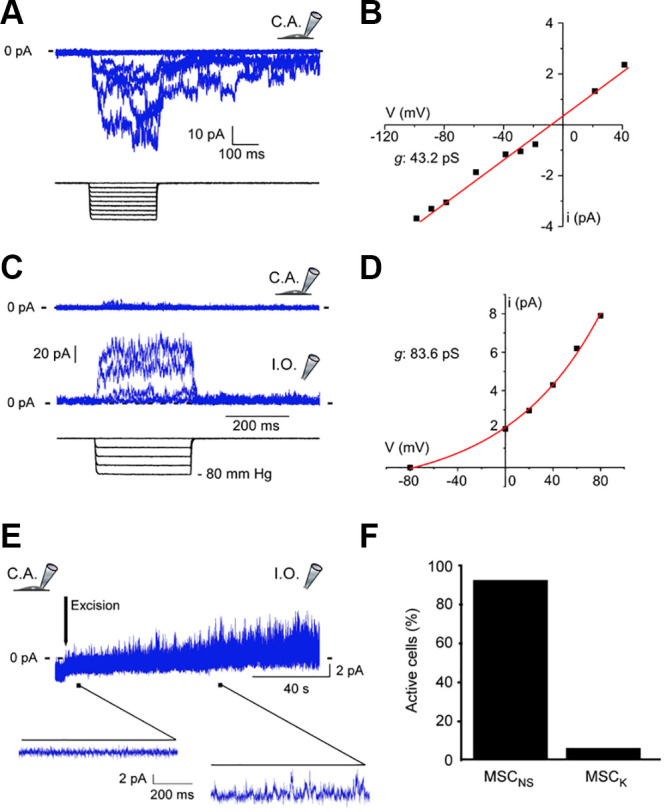
Response of ion channel mechanoreceptors in valvular interstitial cells (VICs). *A*: representative recording of a cation non-selective mechanosensitive channel (MSCNS) in cell-attached patch configuration (C.A.) *B*: current-voltage relation obtained from MSCNS single channel activity at a pressure of −c20 mmHg in the cell-attached configuration for a representative cell. *C*: representative recording of a potassium-selective MSC (MSCK) in cell-attached and in inside-out configurations in response to 500-ms-long negative pressure pulses. *D*: current-voltage relation obtained from MSCK single channel activity at a pressure of -20 mmHg in the inside-out configuration for a representative cell. *E*: the typical progressive activation of MSCK following patch excision. *F*: percentage of cells in which the two types of MSC were recorded. Adapted from Ref. 105, with permission per Open Access terms; see reference for detailed caption.

VECs and vascular ECs sense mechanical stimuli through the glycocalyx on the basal surface of the endothelial cells (FIGURE 13). In response to flow, the glycocalyx has been shown to activate downstream signaling through syndecans to induce cytoskeletal rearrangements for EC elongation. It also induces EC nitric oxide synthase (eNOS) expression via membrane protein glypican for nitric oxide (NO) production (106–111). The shear-induced NO can interact with blood and the underlying VICs to activate several downstream mechanisms (112). It has been shown that the presence of endothelium releasing factors such as NO and endothelin-1 regulate aortic valve stiffness under physiological loading conditions (113). The significance of these changes of aortic valve stiffness in response to stress via paracrine signaling remains to be determined. It has been theorized that remodeling aortic stiffness can optimize stress distributions on the leaflets in a manner that leads to altered function of the VICs. Calcification of the bicuspid aortic valves (BAVs) has been correlated with downregulation of nitric oxide synthase (eNOS) expression (114). Others have also suggested that the lack of local mechanical regulation in BAVs might lead to microtrauma on the endothelial layer, damaging its blood matrix barrier. This may be responsible for the formation of calcification in high-stress areas of the valve (44, 114, 115).

VICs are the major cellular components of the aortic valve that can contract and synthesize extracellular matrix for valvular remodeling. It has been established that the VICs have high plasticity, with the ability to differentiate into other cell types (116). Quiescent VICs are the major resident cells in the valve for maintaining hemostasis of the valve and are fibroblastic in nature (117). However, to perform specific functions, they do have a specific phenotype with the expression of a variety of genes (116, 118–122). Upon damage, disease, or external stimuli, VICs are activated and differentiate into myofibroblasts (123), smooth muscle cells (124), and osteoblasts, thereby remodeling the ECM (125). Other small populations of cells in the valve include smooth muscle cells in the ventricularis (123, 124), progenitor VICs (126, 127) involved in valve repair, osteoblastic VICs, and endothelial/mesenchymal cells that give rise to VICs through endothelial-to-mesenchymal transformation (EMT) (119, 127, 128). Cells from the circulation also contribute to the native VIC population originating from the bone marrow (129, 130) and expressing protein kinase enzyme CD45 (lymphocyte common antigen) (131). VICs respond directly to mechanical stretch with production of collagen and a range of growth factors to induce matrix remodeling (132–134). Whereas VICs are responsible for the maintenance of valvular ECM, the ECM in turn transmits mechanical stimuli between cells. These interactions are predominantly mediated through integrins (135). VICs grown on stiff matrix tend to exhibit the procalcific phenotype with increased transforming growth factor (TGF)β expression, indicating the importance of cross talk in pathogenic processes (117, 136, 137). In addition, aortic VICs express several mechanosensitive ion channels, which include TRPM4, TRPV4, and TRPC6 (105). These showed responses to stretch as well differential expression between normal and calcified valves (105).

**FIGURE 14.**
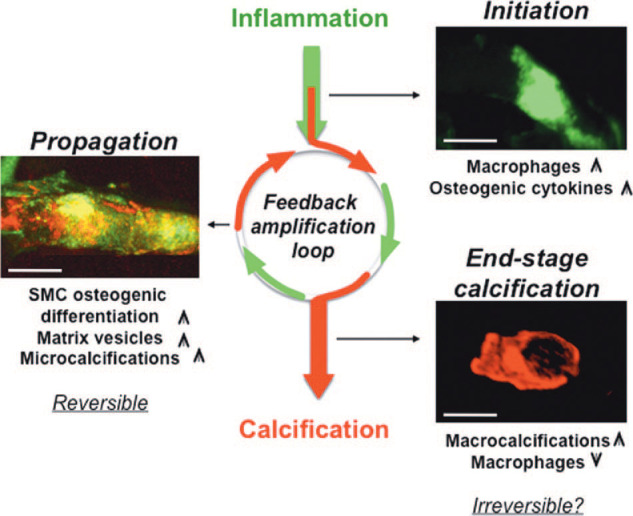
Near-infrared imaging of calcification with the involvement of inflammatory pathways. SMC, smooth muscle cell. Adapted from Ref. 138, with permission from *Circulation Research*.

**FIGURE 15.**
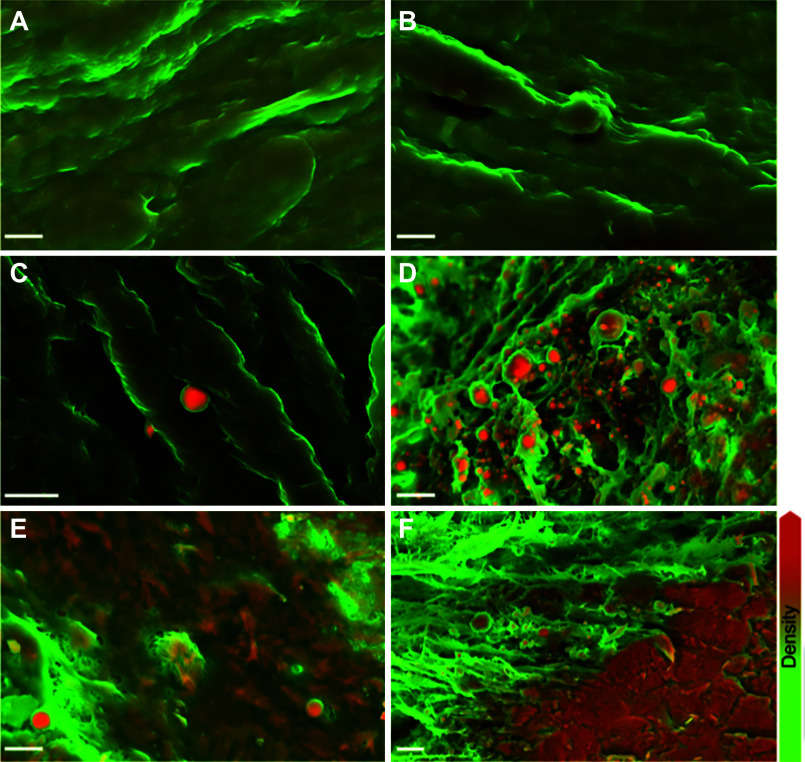
Nano-analytical electron microscopy reveals fundamental insights into the early stages of valve calcification. Structures that are orange are denser materials, whereas green is less dense. *A* and *B*: surface of aortic valve rejected for transplant because of physical damage. Samples lacked macroscopic calcific lesions and were judged not to be atheromic. *C*: surface of aortic valve free from macroscopically observable calcific lesions but presenting dense spherical particles. *D*: calcific lesion on aortic valve with notable dense spherical particles. *E*: calcific lesion on aortic valve, presenting dense spherical particles and fibers. *F*: calcific lesion on aortic valve, presenting dense spherical particles and compact material. Scale bars, 3 µm. Adapted from Ref. 139, with permission from *Nature Materials*.

Aortic valve calcification involves complex cross talk between cells, ECM, and biochemical and biomechanical signals. Although the exact mechanism of initiation is still unclear, it is thought to involve a breakdown in the complex cross talk and dynamism. Partially initiated by abnormal hemodynamic profiles, VICs and VECs undergo differentiation with increased inflammatory signaling, resulting in expression of TGFβs, bone morphogenetic proteins (BMPs), and NO, that leads to further VEC and VIC differentiation toward the osteogenic phenotype. This leads to the morphological changes in the leaflets that create a positive feedback loop that further propels disease progression (21). Inflammatory changes play a major role in initiation of calcification. Early changes of these pathways can be detected by near-infrared imaging (FIGURE 14) (138). Nano-resolution electron microscopy techniques have detected spherical calcified particles, even in valves clinically determined as noncalcified (absence of macroscopic calcific lesions). Additionally, these techniques have revealed that the calcified particles are composed of highly crystalline hydroxyapatite, which exhibits distinct crystallographic and structural differences from bone (FIGURE 15) (139). These observations suggest that aortic valve calcification is a highly dynamic process, requiring an equilibrium to maintain healthy valvular tissue.

### 3.3. Biomechanical and Genetic Factors in Valvulogenesis

**FIGURE 16.**
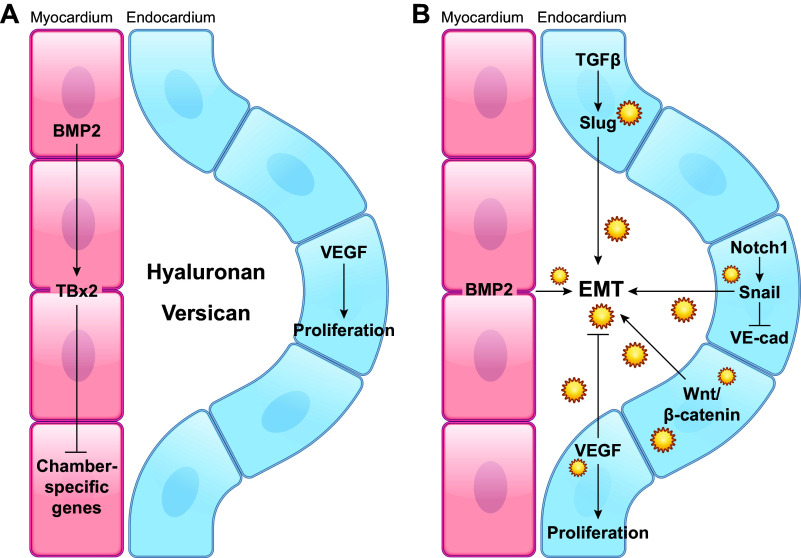
Molecules involved in initiation of valvulogenesis. BMP, bone morphogenetic protein; EMT, endothelial-to-mesenchymal transformation; TGF, transforming growth factor; VE-cad, vascular endothelial cadherin.

**FIGURE 17.**
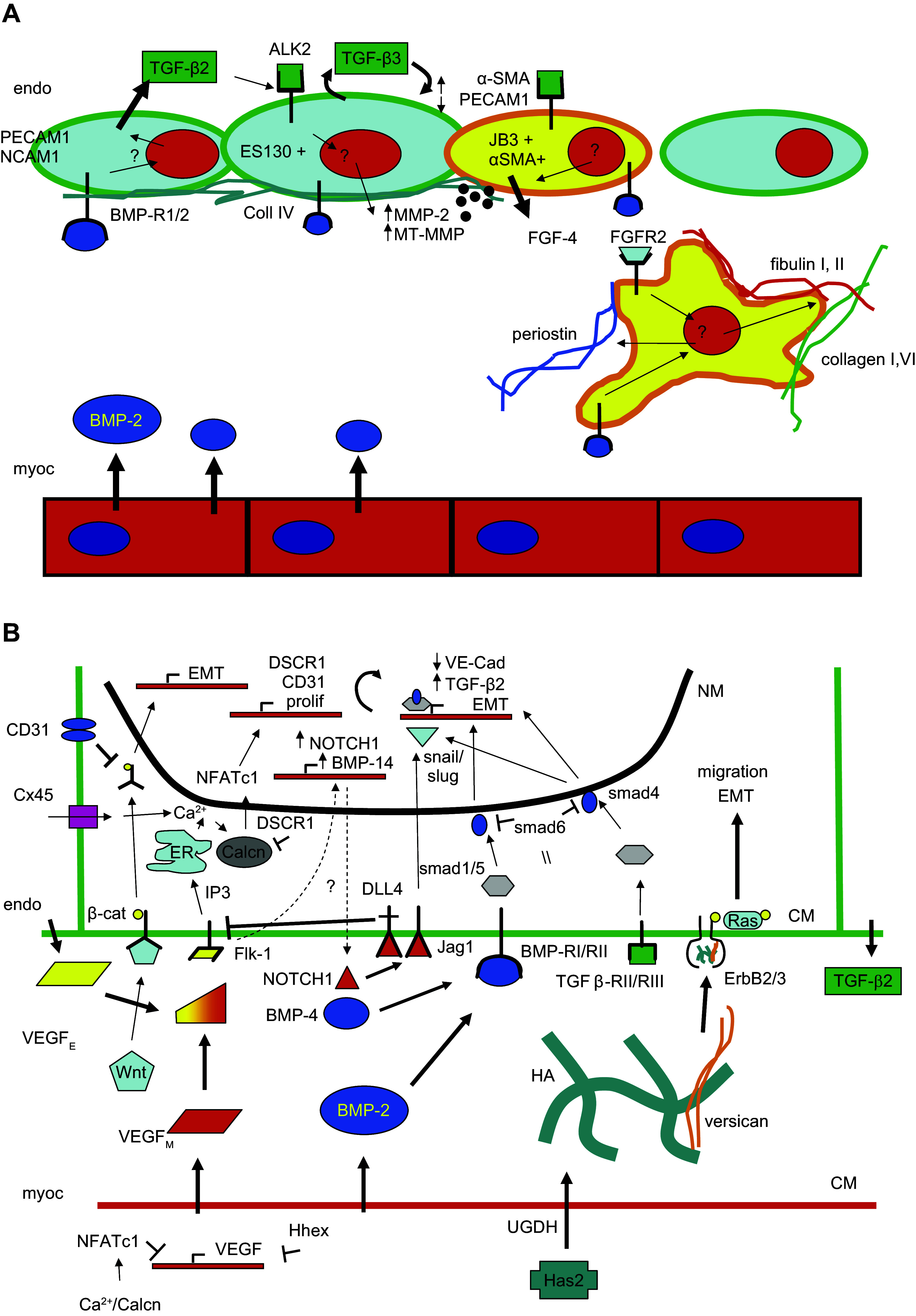
Molecules involved in maturation of valvulogenesis in chick (*A*) and mouse (*B*) models. BMP, bone morphogenetic protein; EMT, endothelial-to-mesenchymal transformation; TGF, transforming growth factor. Adapted from Ref. 141, with permission from *Philosophical Transactions of the Royal Society B*.

Valve development and valvulogenesis is dependent on the interaction between the tightly regulated gene program, including genes by molecules such as NOTCH1, BMP14, and versican (FIGURE 16 and FIGURE 17), mechanical factors (141), and cellular migration, referred to as morphogenesis (141–145). Any interference with or alterations of morphogenesis at a certain time can produce different morphology of the valve of this phenotype (146).

**FIGURE 18.**
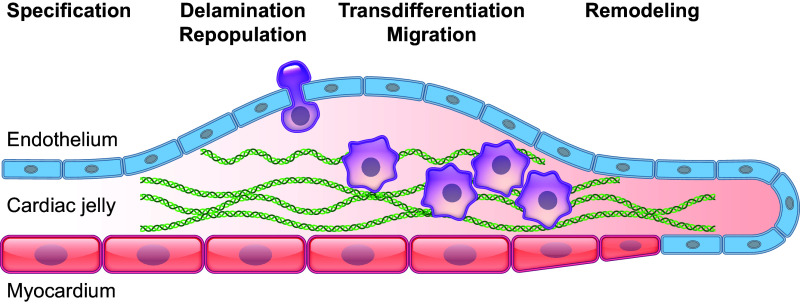
Anatomic overview of heart valve development.

Valvulogenesis is the process of fibrous leaflet formation during embryonic development. There are several mechanical and genetic factors that play important cues to direct valvulogenesis (140, 142, 146–149). The hemodynamic conditions through the heart change both spatially and temporally. This can elicit specific cellular responses during various developmental stages. Although the precise mechanism is still unclear, there are studies showing that the direction and magnitude of wall shear stress affect cardiac morphogenesis. In vivo studies on a zebrafish model have shown that attenuation of flow during heart development prevents endocardial cushion and atrioventricular valve formation (150) (FIGURE 18). *Krüppel-like factor 2* (*KLF2*) has been identified as a key gene in valve development; a knockdown of the gene shows dysfunctional valve development, and its gene expression has been shown to depend upon the presence and magnitude of the reverse flow (151). *KLF2* gene expression has also been shown to regulate fibronectin expression, which is a key ECM component in valve development (152). In a mouse developmental model, *KLF2* induces WNT9b expression, which is required for endocardial-mesenchymal transformation and endocardial cushion formation (153). The precise mechanism of *KLF2* gene sensing of the wall shear stress is still being investigated. It has been shown that the mechanosensitive calcium channels TRPV4 and TRPP2 are expressed in the endocardium during heart valve development and associate with *KLF2* expression (154). Additionally, there are conserved functions for *NFATC1*, Wnt, *NOTCH*, and epidermal growth factor (EGF) signaling in early stages of valve development in zebrafish and mice (148). Others have also suggested that primary cilia, a known player in vascular development, may act as a sensor for shear stress (155, 156).

### 3.4. Morphodynamism

The intricate relationship between morphology and flow has been shown to play a major role in optimizing cardiovascular function (143, 145). The term “morphodynamism” has been used to characterize the interactive relationships between shorelines and the currents in the ocean (157). Near-coastal flow patterns pick up and deposit sand, and the new coastal shape in turn affects flow patterns. The variable time lag required for the sand to be deposited determines the nature of the morphodynamic behavior. Blood vessels adapt to flow patterns in a biologically active manner on both short (vasoconstriction/vasodilation) and long (structural remodeling) timescales, and these adaptations affect subsequent flow patterns. This principle of morphodynamism has been applied in the valve-conserving Yacoub II operation (158).

## 4. FLUID-SOLID BIOMECHANICAL INTERACTIONS

Hemodynamics has an important role in microstructure regulation and cross talk. Furthermore, blood flow is the main force behind the opening and closing of the valve and is deterministic of valve function. Simultaneously, the structure of the AVA influences the fluid dynamics, a close intertwined play leading to a complex two-way fluid-structure interaction that is crucial for full AVA function. Studying the fluid mechanics and the fluid-structure relations in depth is essential for correctly understanding and characterizing the physiology and function of the valve.

### 4.1. Valve and Flow Relations

The movement and function of the valve are closely linked to the blood flow around it. The transvalvular pressure (TVP) is the moving force behind the opening and closing of the valve, which is captured through the nonuniform pressure distribution on the leaflets. When the valve is closed the pressure difference across the valve is balanced by the extensive forces in the leaflet, which are transmitted to the aortic wall through the valve attachments at the commissures through the collagen fibers (46, 160). The fibrous body in the aortic wall certainly plays a part in supporting the relatively large forces that are involved. The maximum pressure acting on the aortic side of the leaflets can be >120 mmHg (15 kPa) (161), and the aortic cross-sectional area is on the order of 4 cm^2^ (162), which means that the axial force is on the order of 6 N.

When the transvalvular pressure difference reverses at the start of systole, the tensile stress in the leaflets is relieved and the membranelike leaflets are prone to buckling. Once they start to open, the mechanics change dramatically (39). The density of the leaflets is very similar to that of blood, so they move in concert with the adjacent fluid, except that they can still bear in-plane stresses and they do not allow blood to cross them (163–167). During this part of the cardiac cycle, it might be more accurate to say that the leaflets are displaced by the moving blood rather than moving under the influence of the transvalvular pressure differences. This interaction of fluid flow and finite deformation of a pliable structure, similar to a sail flapping in the wind, is quite complex to analyze. This is the domain of computational modeling referred to as FSI.

The relationship between the valve’s structure and the blood flow through it is observed when comparing the 3-D flow characteristics of bicuspid to trileaflet valves and rigid to flexible leaflets (12, 168). In vitro and in vivo magnetic resonance imaging (MRI) studies of asymmetric bicuspid valves show significant alteration to turbulent flow and increased wall shear stress through the BAV and ascending aorta (168–170). An increase of wall shear stress can lead to aortic wall degradation, aortic aneurysm, and aortic dissection (171, 172). In addition, the observation of an enhanced rate of ascending aortic stiffening in BAV patients (173) also supports the theory that changes in hemodynamics, flow, and wall shear stress in BAV patients are often associated with aortic wall pathology and dilation.

### 4.2. Impact of AVA on Blood Flow

**FIGURE 19.**
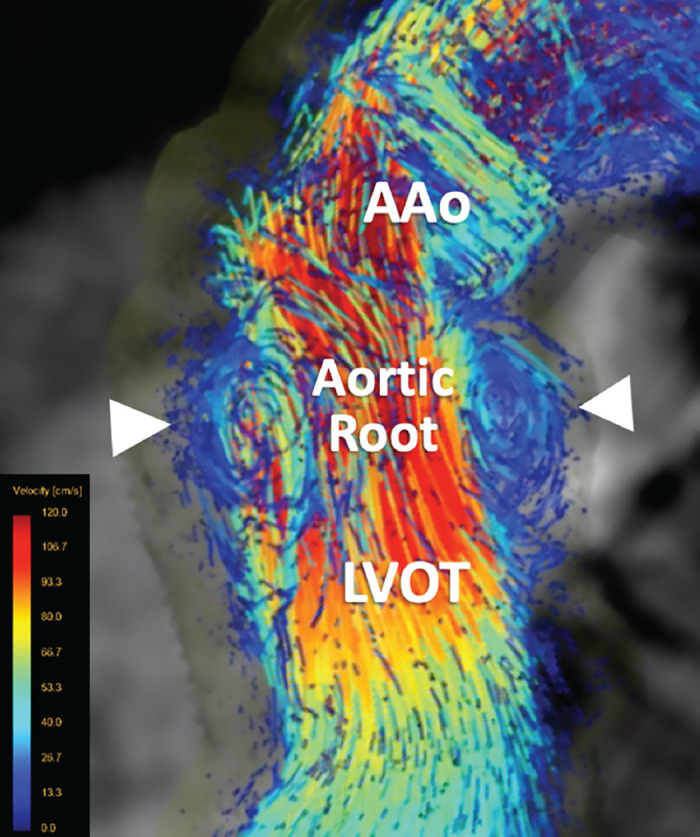
Four-dimensional (4-D) flow MRI image showing the physiological recirculating vortical flow in the aortic root of a healthy subject. Adapted from Ref. 159, with permission per Open Access terms. The white arrowheads show the vortices in the sinuses of Valsalva. AAo, ascending aorta; LVOT, left ventricular outflow tract.

The root enables the blood flow to form vortices in the sinus (FIGURE 19), which in turn feed the coronary arteries and aid in valve closure. The sinus geometry, aided by systolic vortices and elastic expansion with systolic pressure, prevents leaflets from contacting the walls of the sinus, which might block coronary flow and create closure problems due to leaflet adherence. The geometry of the sinus creates a space between the aortic wall and its corresponding open leaflet. Because of the shape, this space favors the formation of eddies behind the leaflets, which holds them away from the aortic wall. The eddies are also important in promoting valve closure. After peak systole, the currents force the leaflets to move back away from the aortic wall so that they almost totally coapt before the end of systole (64). Upon valve closure, the sinus provides a wide berth that serves as a reservoir for coronary perfusion (64), which happens mainly in diastole because of reduction in distal resistance associated with myocardial relaxation (174).

Moreover, the sinuses have a role in stress reduction on the leaflets. The vortex structures that form within the sinuses after the onset of systole play a major role in ensuring a homogeneous valve closure (100). Studies comparing blood flow patterns and stresses in aortas with and without the sinuses demonstrate that their presence facilitates smooth valve closure. This homogeneous and smooth motion relieves the abnormal stresses building on the leaflets (175). The sinuses can be seen as the basic structural and functional unit of the AVA, although further importance has been given to the function of the interleaflet triangles, which are said to allow the sinuses to act independently and are therefore crucial to proper valve function (64).

### 4.3. Vortices in Sinuses of Valsalva

The aortic sinuses play an important role in valve dynamics and blood supply to coronaries (176–181). When aortic valve flow is simulated in the absence of sinuses, the leaflets close less efficiently because of the absence of the natural flow patterns in the sinuses. The sinuses have a major function of enabling vortex formations throughout systole that also facilitate the reattachment of the main aortic jet at the STJ (176–184) (FIGURE 19). The spatiotemporal resolution of the vortices and countervortices in the sinuses results in coupling to the kinematics and oscillation of the leaflets (185), which illustrates the importance of in vitro studies in understanding the sophisticated functions of the aortic valve apparatus.

**FIGURE 20.**
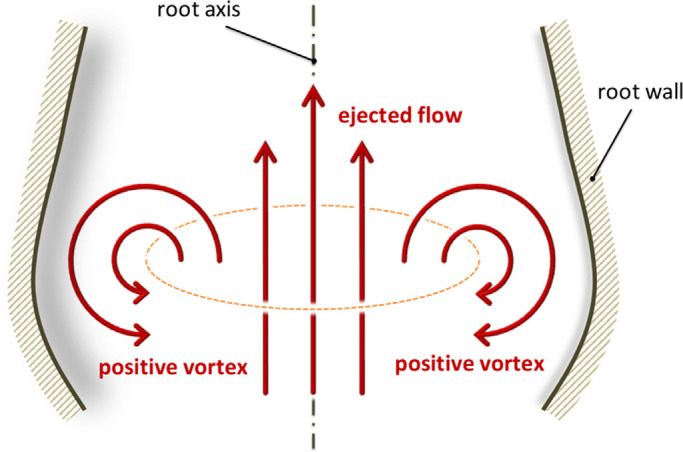
Bellhouse's early suggestion describing the formation of a vortex ring during aortic flow ejection. Adapted from Ref. 176, with permission per Open Access terms.

Early publications of Bellhouse (182–184) suggested that the sinuses “trap” the vortices generated in systole, preventing the valve leaflets from impinging on the wall and helping the leaflets close as flow decelerates (FIGURE 20). This was accepted as common knowledge in the early days of aortic mechanics. Subsequent numerical simulations (175, 186–189) and MRI velocimetry (190–194) appeared to confirm this theory, although more recent simulations and experimental methods have cast doubt (176, 195).

The fault in Bellhouse’s theory is the extension of flow patterns imagined, measured, or calculated in two dimensions to the fully 3-D situation. This is compounded by the fact that two-dimensional (2-D) slices or projections of 3-D flow patterns can resemble the 2-D approximations, which in this case led others to perpetuate Bellhouse’s theory. By Stokes’s theorem, vortex lines cannot end within the fluid, which means that in 3-D flow they must eventually form a closed loop (topologically equal to a circle). 2-D vortex lines can end on solid boundaries or at infinity (196, 197), and therefore extrapolating 2-D results to three dimensions is ill-advised.

### 4.4. Aortic Root Dynamics and Impact on Flow

The ascending aortic tissue is capable of undergoing large strains of 15% or more in response to the pressure pulse. The elasticity of the entire aorta and its largest branches is a large component of the compliance element of the windkessel function. The windkessel effect converts the phasic systolic inflow produced by ventricular ejection into a smoother, more continuous blood outflow to peripheral vessels (198). The aorta stores ∼50% of the left ventricular stroke volume in systole, which is then propelled into the coronary arteries and other distal vessels in diastole, known as the reservoir function (65). The storage of elastic potential energy while blood volume accumulates in the aorta also has an influence on the left ventricular afterload (65). Interventions to treat aortic valves should aim to maintain a healthy and normal windkessel function to maintain healthy elastic tissue and reservoir function.

Aortic root kinematics inside the chest have been suggested to have an impact on flow (92, 199). The kinematics assessed with landmark point tracking on computed tomography (CT) images of the aortic root has shown a peak acceleration of 10 m/s^2^ at the time of valve closure, implying an aortic root movement along its axis away from the LV. The aortic root accelerations could explain the presence of the dicrotic notch pressure at the time of valve closure. The sudden rise in aortic pressure at the time of valve closure is a counterintuitive phenomenon, as the flow velocities are diminishing. However, the sudden positive acceleration from the aortic root could be the main reason behind the sudden rise in pressure at the time of valve closure, analogous to a water hammer (200).

Moreover, the annulus temporal deformation showed a consistent radial contraction from the right coronary sinus across to the right posterior interleaflet triangle, which is located between the noncoronary sinuses and left coronary sinuses (LCS) (201). Porcine aortic roots exhibit a rotation of the root toward the left posterior interleaflet triangle, which is between the right and left coronary sinuses, during the cardiac cycle (98, 99, 202), referred to as active dynamism (44).

### 4.5. Aortic Root and Coronary Flow Interaction

**FIGURE 21.**
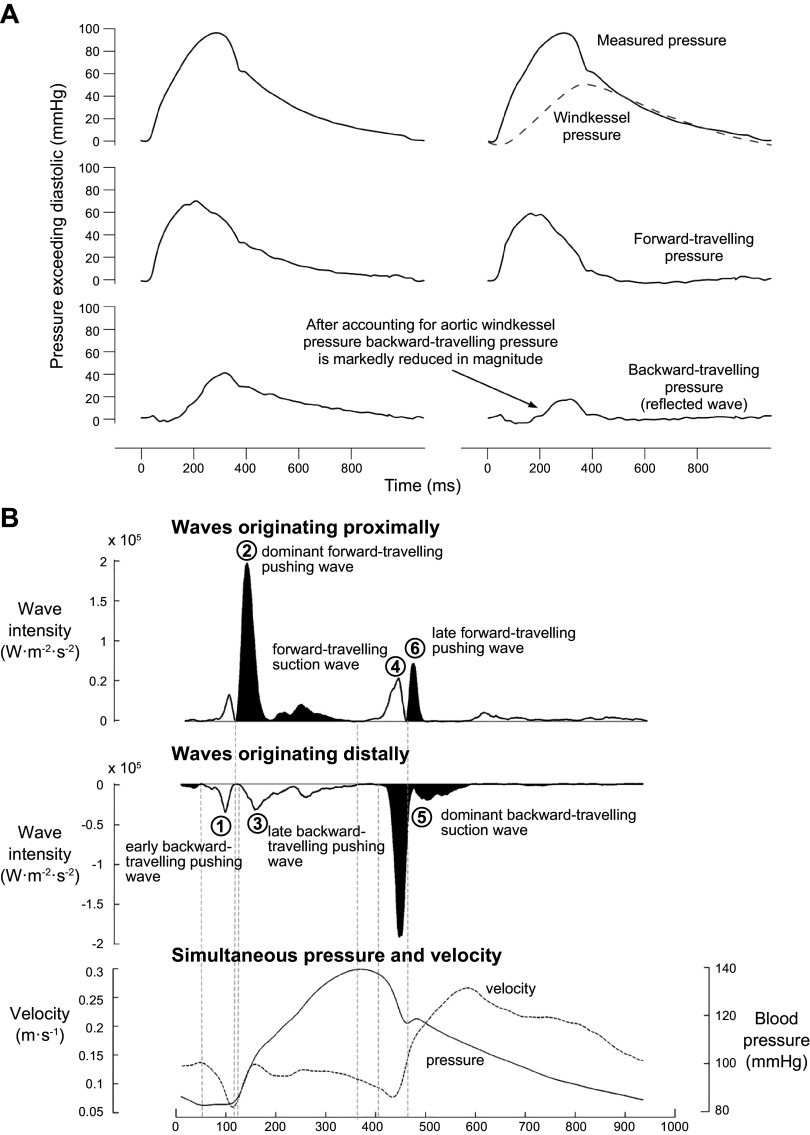
*A*: the relationship between aortic pressure and forward and backward pulse waves. Adapted from Ref. 205, with permission from *Heart*. *B*: wave intensity analysis in healthy unobstructed circumflex artery. *Top*: forward waves originating near the aorta. *Middle*: backward waves originating near the microcirculation. *Bottom*: total pressure and velocity waveforms used in the calculation of wave intensity analysis. Adapted from Ref. 206, with permission from *Circulation*.

**FIGURE 22.**
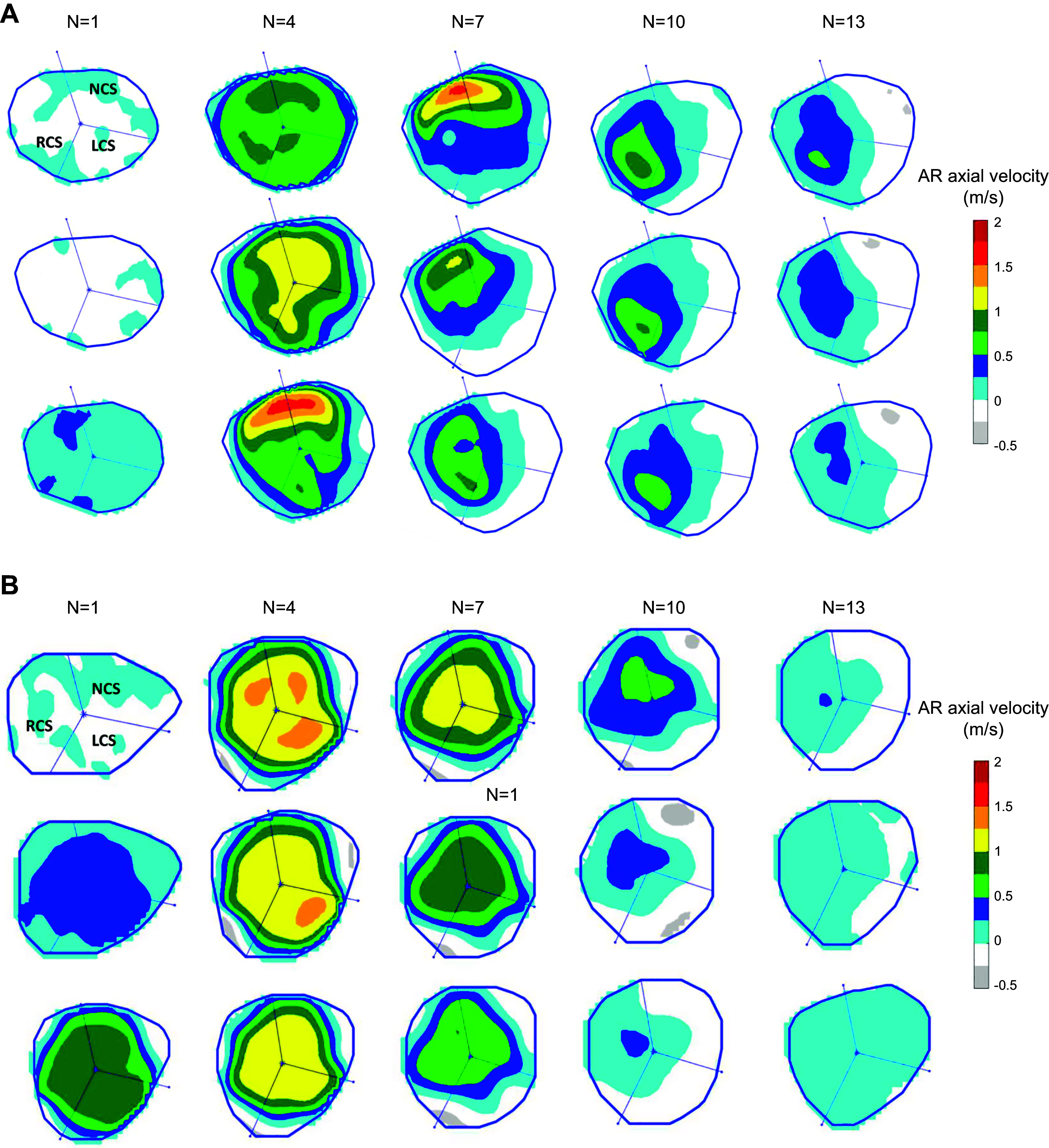
Aortic root (AR) hemodynamics in the 3 sinuses of Valsalva using the axial velocity flow mapping. *A*: in a hypertrophic obstructive cardiomyopathy (HOCM) patient. *B*: in a control subject. LCS, left coronary sinus; *N*, cardiac frame number; NCS, noncoronary sinus; RCS, right coronary sinus. Adapted from Ref. 207, with permission per Open Access terms.

**FIGURE 23.**
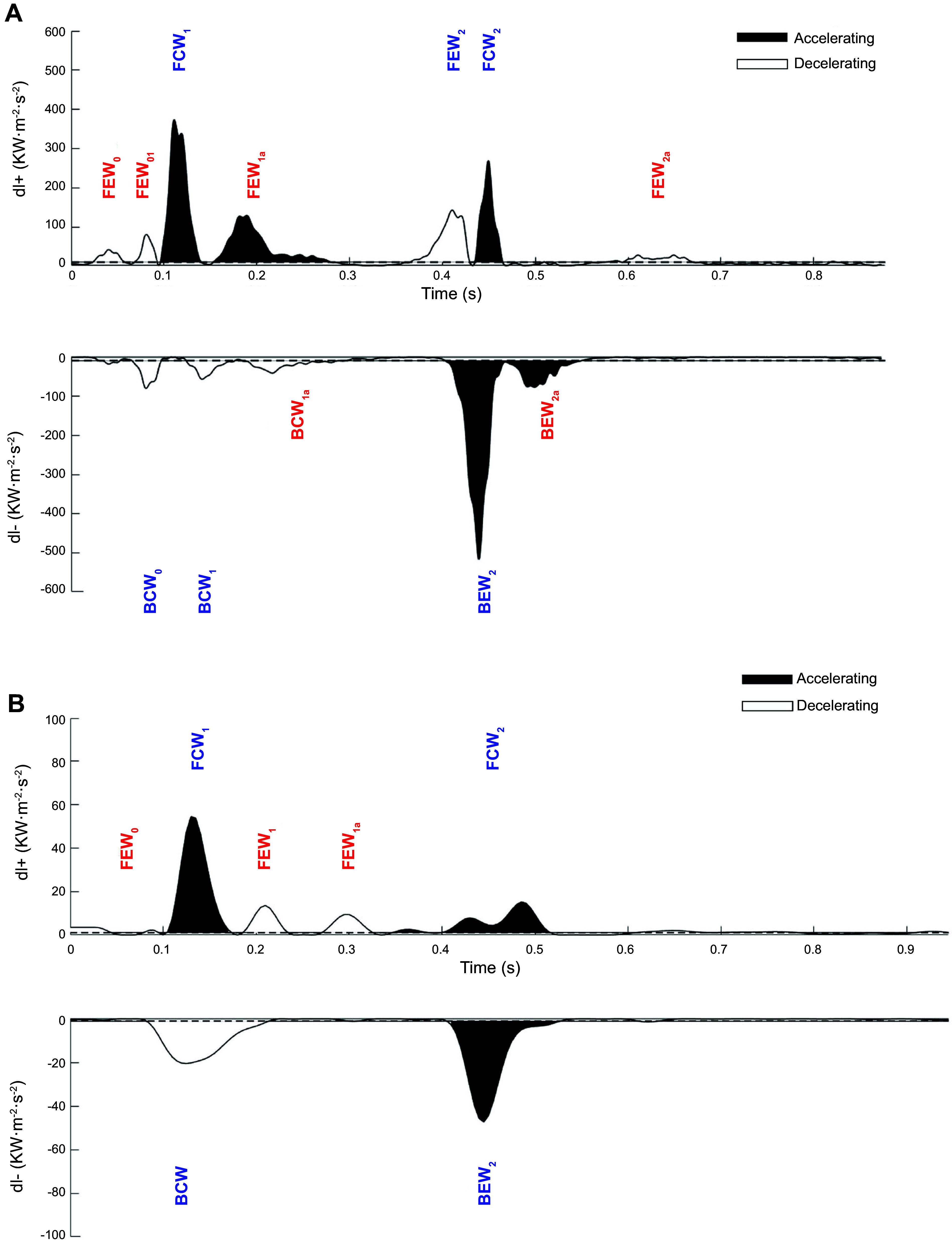
Identification of significant wave intensity peaks using maximum entropy method. *A*: control subject. *B*: hypertrophic obstructive cardiomyopathy patient. dI+ is the forward ensemble average waveform. dI− is the backward ensemble average waveform. The blue colored waves are the classical dominant waves, and red colored waves are the secondary new waves. BCW, backward compression wave; BEW, backward expansion wave; FCW, forward compression wave; FEW, forward expansion wave. Adapted from Ref. 208, with permission from *Frontiers in Cardiovascular Medicine*.

The pattern of flow in the aortic root plays a role in the regulation of coronary blood flow and its wave intensity analysis [WIA; used to study the dynamics of cardiovascular blood flow throughout the cardiac cycle through pressure and velocity (203, 204)]. This relationship was first pointed out by Justin Davies and Kim Parker (FIGURE 21) (205, 206). Further work by Nadine Francis and colleagues served to clarify this relationship, where it was evaluated in both normal and hypertrophic cardiomyopathy (HCM) (207). The patterns of flow in the three sinuses of Valsalva were equal in the normal root, in contrast to the vortices in the LCS, which were markedly perturbed in HCM (FIGURE 22). This resulted in what was considered perturbed coronary hemodynamics reflected in atypical new waves in coronary WIA (FIGURE 23) (208). Thus, the complex relationship between the various instantaneous vortices in the aortic root during the cardiac cycle could influence coronary flow (207), yet the causality and the exact mechanisms behind it have not been determined and thus should be studied further.

### 4.6. Leaflet Kinetics and Kinematics

In vivo studies of the aortic valve opening show active dynamism, with the valve opening before any change in pressure or flow (209). In addition, the function of the AVA is a result of a two-way interaction between structure and flow. The leaflets’ opening and closing motion is dependent on the blood flow, and the flow patterns are in turn affected by the structure’s shape, viscoelastic properties, and active contractile behavior. This means that the pressure distribution on the leaflets is dependent not only on its instantaneous shape but also on its motion (12). At the boundaries of the blood and AVA, interaction forces determine local accelerations, while displacements are equal (to keep the two in contact). The complexity of these interactions is analogous to analyzing the bouncing of a ball on a trampoline compared to bouncing it on a rigid floor.

The net forces on the valve leaflets resulting from transvalvular pressure (TVP) drive the opening and closing of the valve. The TVP is nominally a few millimeters of mercury in systole and equal to diastolic pressure in diastole, under normal conditions. However, fluid is in continual motion on both sides of the leaflets, implying that there are local differences in pressure (a necessity for fluid motion). A proper analysis of leaflet dynamics should account for these spatial pressure variations (12). The resulting leaflet motions influence the flow patterns. This is the domain of FSI, a branch of computational mechanics summarized below. These techniques can reveal unprecedented details on the three-dimensionality of the flow (210–213), which has also been shown in in vitro studies (183, 214–216). The strong coupling between the leaflets and the surrounding blood flow, the existence of a central jet emanating from the valve orifice, and vortical structures developing in the sinus cavity all influence valve function (217). These modeling strategies have been further applied to understand differences in hemodynamics associated with normal tricuspid AV versus BAV, where in the normal AV the aortic jet goes straight through the valve along its centerline versus in BAV, where the jet is redirected to the aortic wall (218, 219).

### 4.7. Ventriculo-Arterial Coupling

**FIGURE 24.**
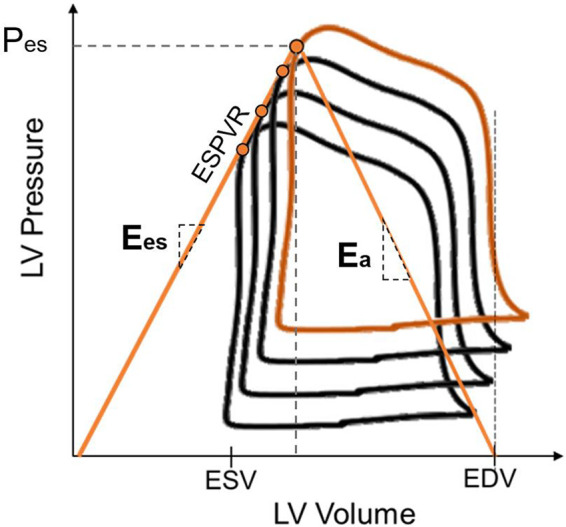
Illustration of elastances obtained from pressure-volume (PV) loops. *E*_a_, arterial elastance, obtained as the slope connecting the end-systolic point on the PV loop to the end-diastolic volume (EDV) at 0 pressure; *E*_es_: end-systolic elastance, obtained as the slope of the end-systolic ventricular pressure-volume relation (ESPVR); ESV, end-systolic volume; LV, left ventricle; P_es_, end-systolic pressure.

An efficient anatomical and functional relationship between the LV, the AVA, and the aorta is necessary to ensure sufficient blood flow in the vasculature and perfusion to all organs. (220). Given that ventricles and arteries are linked both anatomically and functionally, the concept of ventriculo-arterial coupling (VAC) was developed to describe the energy transfer between ventricular contractility and arterial afterload. It allows the assessment of the performance and efficiency of the cardiovascular system, in both health and disease. Sunagawa et al. (221) described ventricular contractility using the pressure-volume (PV) loop’s end-systolic elastance (*E*_es_), i.e., the slope of the end-systolic ventricular pressure-volume relation (ESPVR), and the arterial afterload as the arterial elastance (*E*_a_) (FIGURE 24). This arterial elastance represents the load the ventricle needs to pump against. Coupling is therefore calculated as the ratio of *E*_a_ to *E*_es_. The first description of the ventricular PV relation was published by Otto Frank in the nineteenth century. The PV relation thus became a reference tool to assess the efficiency of the ventricle pumping function (222).

A healthy coupling, i.e., an efficient energy transfer, has a value of around 1. Uncoupling occurs when the *E*_a_ exceeds the *E*_es_, meaning that the coupling will be larger than 1. This results in a compromised function and could lead to heart failure (222, 223).

Any abnormality in the anatomical or functional relationship of the LV, AVA, and aorta has an adverse effect on the entire function of the cardiovascular system (220). To maintain a healthy VAC value in light of those abnormalities (diseases), compensatory mechanisms take place. If the afterload is increased, for example as in cases of aortic stenosis (AS), the LV myocardium thickens to be able to pump more blood (224). This leads to a reduced stroke volume since the ventricular cavity decreases in size. To compensate for this decrease in volume, the ventricle dilates (220). With advanced severity of AS, *E*_a_ increases and *E*_es_ decreases, which leads to uncoupling. In cases of aortic valve regurgitation, there is a volume overload in the LV. In the beginning of the condition, the system is coupled because of an increase in LV contractility to accommodate for the increased volume. However, as the condition is maintained, the contractility drops and the stroke volume is reduced. Consequently, the VAC increases and the system becomes uncoupled (224). Furthermore, the coupling ratio right after a transcatheter aortic valve replacement (TAVR) procedure was shown to be significantly associated with the prognosis at follow-up (225).

## 5. METHODS/TOOLS TO STUDY THE AVA

There are several tools available for clinicians and researchers to study the AVA’s structure-function relations, dynamism and cross talk, blood flow, and the solid-fluid interactions, both quantitatively and qualitatively. Medical imaging techniques are used for clinical evaluations and assessment, and more recently computational modeling is being explored for more in-depth insights and for its abilities to predict clinical outcomes.

### 5.1. Imaging Techniques

**FIGURE 25.**
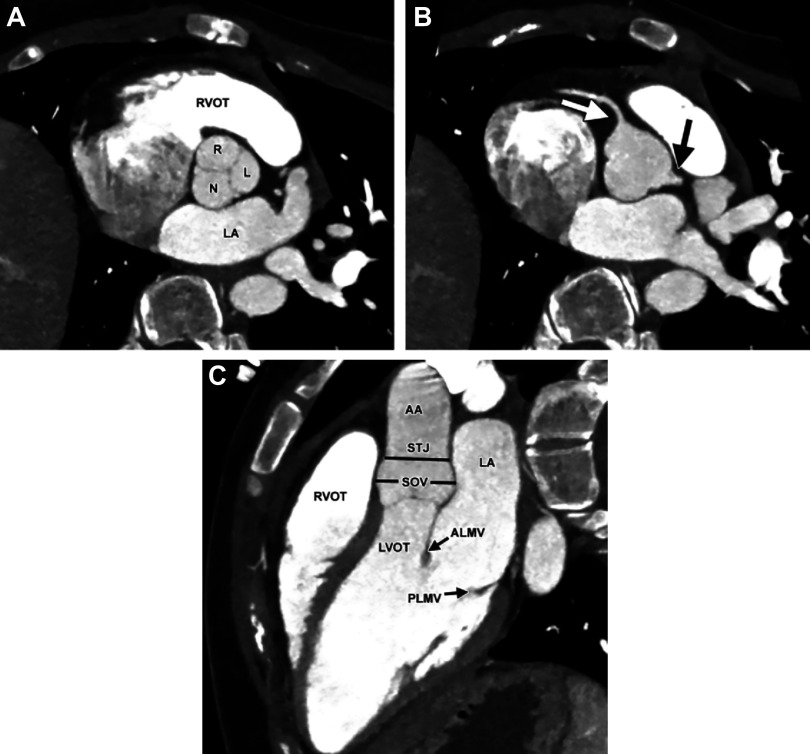
Normal aortic valve (AV) apparatus (AVA) anatomy by cardiac computed tomography (CT) imaging. *A*: double oblique CT image at the AV level with right (R), left (L), and noncoronary (N) cusps. LA, left atrium; RVOT, right ventricular outflow tract. *B*: double oblique CT image at the coronary ostia level. The white and black arrows point to the coronary arteries at their ostia. *C*: long-axis cardiac CT image showing a 3-chamber view. AA, ascending aorta; ALMV, anterior leaflet of the mitral valve; LVOT, left ventricular outflow tract; PLMV, posterior leaflet of the mitral valve; SOV, sinuses of Valsalva; STJ, sino-tubular junction. Adapted from Ref. 226, with permission from *RadioGraphics*.

Currently, imaging is being extensively used as an evaluation and diagnosis technique to assess AVA function. Ideally, medical imaging should provide quantitative information on blood flow and tissue displacements at high spatial and temporal resolution to aid diagnosis and treatment. In reality, there are trade-offs. The main imaging modalities are computed tomography (CT), echocardiography (ECHO), and magnetic resonance imaging (MRI). As CT images have a high spatial resolution (see FIGURE 25), it can capture the structure in detail and is therefore widely used to assess leaflet stenoses and calcifications (226). However, it has a low temporal resolution and does not provide any information concerning the flow. ECHO and MRI, both 2-D and four-dimensional (4-D), images are capable of providing measurements of the flow, including visualization of vortices (192, 227–229), helical flow patterns in the aorta (230), and velocity data for model-based calculations of wall shear stress (231). A comparison of these modalities is given in TABLE 1.

**Table 1.** Comparison of imaging modalities for assessing AV

Image Modality	Transthoracic Echocardiography (TTE)	Transesophageal Echocardiography (TEE)	Computed Tomography (CT)	Magnetic Resonance Imaging (MRI)
Spatial resolution	Pixel size: 1–2 mm	Pixel size: 0.5–1 mm	Pixel size: 0.6–0.75 mm	In-plane resolution similar to CT, but through-plane resolution is 6–8 mm
Temporal resolution	30–60 frames per second	30–60 frames per second	With ECG gating, 10–20 frames per second	With ECG gating, 20–30 frames per second
Flow velocity and volume flow rate measurements	Excellent; Doppler US commonly used for flow measurements	Excellent; Doppler US commonly used for flow measurements	No current clinical method for flow measurements	Good; cine phase-contrast imaging used for flow measurements
Patient-specific limitations	Scattering and attenuation limit assessment in some patients	Invasive technique, requires sedation	Need for radiation and contrast material injection	Cannot be used on patients with metallic implants

See also Ref. 226. US, ultrasound.

**FIGURE 26.**
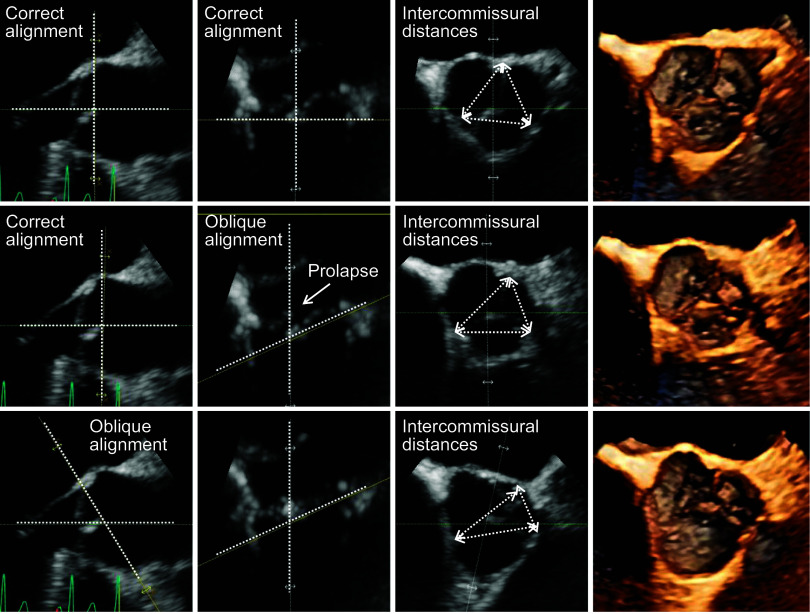
The use of two (2-D)- and three (3-D)-dimensional echocardiography to perform aortic valve apparatus (AVA) measurements, such as the intercommissural distances. Adapted from Ref. 232, with permission per Open Access terms.

ECHO has a high temporal resolution, which can be used to closely study the leaflet mobility and valve kinematics. 2-D and 3-D echocardiography provide valuable quantitative data of the anatomy and function of the AVA (232) (FIGURE 26). They are also widely used as a key tool in noninvasively diagnosing and evaluating the severity of aortic stenosis (233). There are multiple techniques to evaluate the function of the aortic valve with Doppler-echocardiography. Among those are the use of the measured aortic jet maximum velocity, the mean pressure gradient deduced from velocity measurements across the valve orifice, or the measured aortic valve area, which can also be used to assess function. The main limitation when using echocardiography images is that they are subject to user error: if the sound beam used is not parallel to the aortic stenosis velocity jet, the results will not be accurate. Also, measures such as the aortic valve area assume that the aortic valve orifice is circular, which is not necessarily the case. Finally, Doppler-echocardiography does not allow for a high resolution of the flow field and the vorticity pattern (234).

**FIGURE 27.**
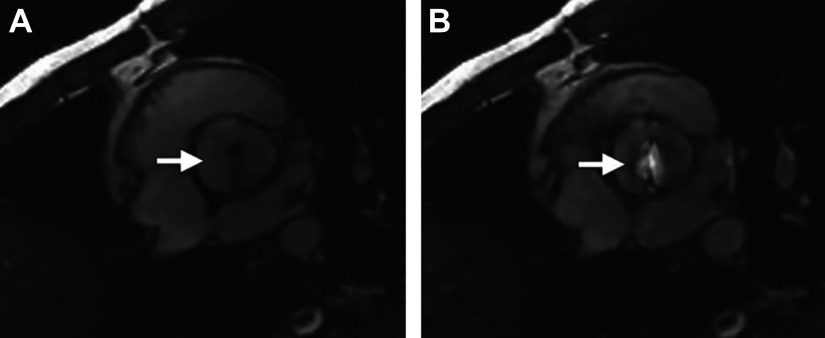
Magnetic resonance (MR) image of bicuspid aortic valve (BAV), with fused right and non-coronary cusps (arrow), with stenosis and dilation obtained with ECG gating in a plane parallel to the AV during diastole (*A*) and systole (*B*). Adapted from Ref. 226, with permission from *RadioGraphics*.

**FIGURE 28.**
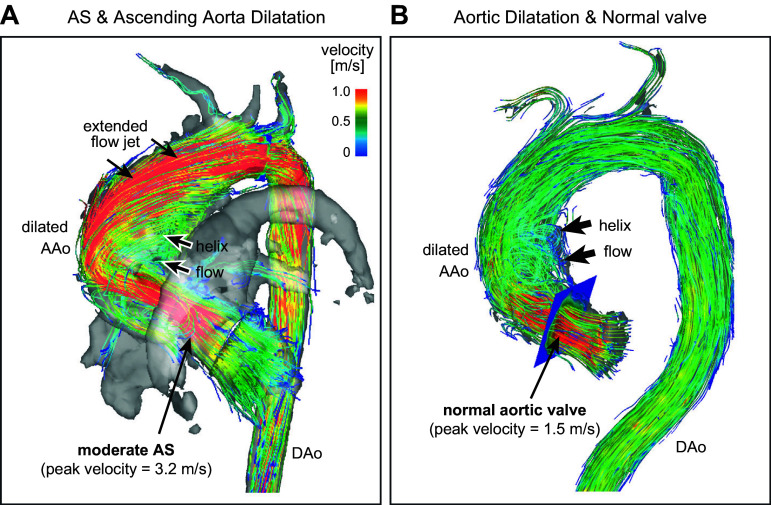
Visualization of instantaneous blood flow velocity streamlines from 4-dimensional (4-D) flow MRI for moderate aortic stenosis (AS) with a dilated ascending aorta (AAo) (*A*) vs. normal aortic valve with a dilated ascending aorta (*B*). DAo, descending aorta. Adapted from Ref. 236, with permission from *JACC: Cardiovascular Imaging*.

**FIGURE 29.**
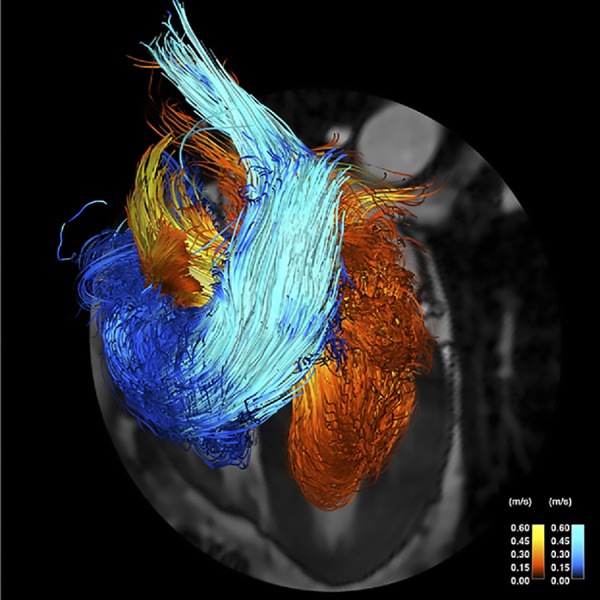
Pathline visualization of cardiac blood flow with particle tracking in cardiac magnetic resonance (CMR). It reflects the temporal dynamics of the 3-dimensional (3-D) blood flow throughout the cardiac cycle. Yellow pathlines are for the trace of blood flow from the left atrium to aorta. Blue pathlines are for the trace of blood flow from the right atrium to pulmonary arteries. Adapted from Ref. 228, with permission from *JACC: Cardiovascular Imaging*.

MRI has both good spatial and temporal resolution. However, it does not capture the leaflets well (FIGURE 27), but it does capture the blood flow more clearly than ECHO. Factors such as the peak aortic jet velocity, deduced mean pressure gradient across the valve, aortic valve area, and regurgitation can be derived from 2-D MRI. The multidirectional velocity encoding provided by 4-D MRI technology also allows the complete 3-D visualization of the flow through the heart and aorta (235) (FIGURE 29). Quantitative analysis of systolic vortex formation behind aortic valve cusps and the helical flow in the ascending aorta can be achieved with 4-D flow images, as seen in FIGURE 28 (192). One of the limitations of 2-D MRI compared to ECHO is that phase-contrast acquisitions have lower temporal resolution than echo Doppler-based methods, which may lead to underestimation of peak velocity (234). 4-D flow has also been shown to not be accurate near walls. It has been shown that although MRI and computational fluid dynamics (CFD) show the same wall shear stress (WSS) distribution, the magnitudes are underestimated in MRI (237).

### 5.2. Energy Loss

It is also important to consider energy conversion and loss when assessing AVA function. Although the term “energy loss” is often used in this context, it is worth noting that energy is never really lost but rather just converted to another form. Throughout the circulation process, mechanical energy is converted between pressure (work performed to move fluid), potential energy due to gravity, and kinetic energy in the form of velocity (238). This is shown in the steady Bernoulli’s equation (Eq. *1*), relating pressure, gravitational energy, and kinetic energy (velocity) between two points, *1* and *2*, along the flow path (i.e., streamlines for steady flow), assuming no viscosity:

(*1*) $$\frac{1}{2}\rho v^{2}+\rho gh+p=\text{constant }(\text{along a flow path})$$

where ρ is fluid density, *v* is magnitude of the fluid velocity vector, *h* is height opposite the direction of gravitational acceleration (*g*), and p is pressure.

The primary source of mechanical energy in the systemic circulation is the work done by the LV during contraction. Mechanical energy can also be converted into heat due to friction between the moving blood and the stationary vessels or between the flowing blood and itself because of its viscosity (i.e., kinetic energy of the fluid converted to thermal energy). This energy cannot be recovered back into the forms listed above and therefore is considered to be “lost.” Frictional energy losses can be categorized as viscous losses, turbulence losses, or flow separation losses (238). Nonetheless, in all cases frictional energy losses are due to viscosity; turbulence leads to viscous losses due to energy exchange between eddies, and flow separation leads to turbulence.

Although energy loss is an intrinsic feature of the circulation, as the LV is designed to contract and provide the needed energy to overcome the losses inherent in delivering the blood to peripheral tissues (238), pathologies of the AVA causing stenosis, regurgitation, or aneurysms increase the irreversible energy loss. It is therefore clinically relevant to compare levels of energy loss between normal and diseased subjects and identify the locations of increased loss to identify problem areas.

When the blood goes from the LV into the aorta through the AVA, it is first accelerated from the LVOT to a vena contracta. However, as the flow goes through the valve it is decelerated because of the divergence downstream of the vena contracta. This conversion from dynamic pressure into static pressure is referred to as the pressure recovery. Negative pressure gradients associated with pressure recovery can enhance instabilities in the fluid and trigger turbulence, and thereby dissipate more energy. For healthy valves, this loss of energy is negligible. However, as the extent of pressure recovery is related to the ratio between the valve’s EOA and the cross-sectional area of the ascending aorta, the energy loss can become significant in cases of stenosis and some prosthetic valves, which would lead to overloading the LV (239). The energy loss index (ELI), introduced by Pibarot et al. (240), is used as a tool for stenosis assessment and clinical decision-making as it takes into account the pressure recovery phenomenon.

**FIGURE 30.**
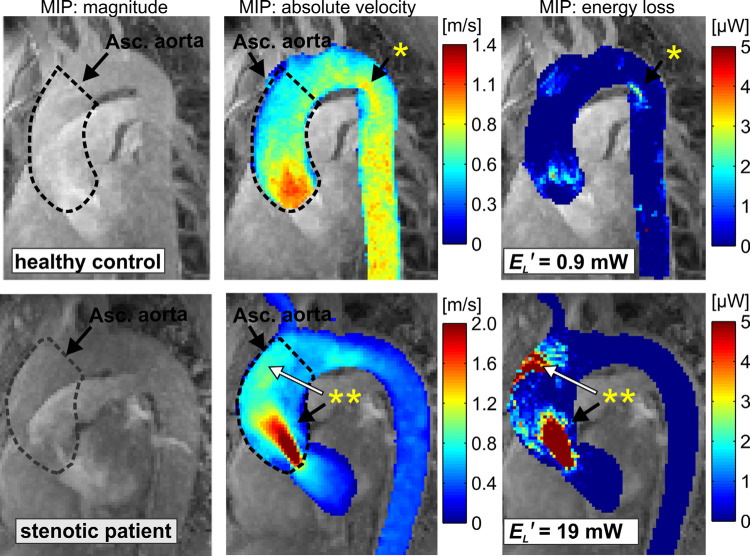
Four-dimensional (4-D) flow MRI images showing the velocity field and energy loss through the aortic valve and aorta. E_L_′, cumulative peak systolic energy loss in the ascending aorta. Adapted from Ref. 241, with permission from *Magnetic Resonance in Medicine*. Arrows, *, and ** are from source image and have no significance in this review.

An unhealthy aortic valve function, due to stenosis or BAV for instance, is also associated with an alteration of the postvalvular hemodynamics: helical flow, increased vorticity, and WSS (171). These disturbances in the flow pattern lead to higher energy loss. Therefore, another important indicator of valve function is the viscous energy loss in the ascending aorta, which corresponds to the mechanical kinetic energy irreversibly converted to thermal energy as a result of frictional forces relating to the fluid viscosity and no‐slip condition at the walls. This novel marker can be estimated noninvasively with 4-D flow MR imaging and allows the description of altered hemodynamics. This marker can quantify differences in energetic loss in patients with aortic dilation and aortic valve disease. Normal values for ascending aorta viscous energy loss are ∼1.2 ± 0.6 mW, whereas this value increases for diseased aortas and aortic valves, i.e., 2.2 ± 1.1 mW and 10.9 ± 6.8 mW for dilated aortas and aortic stenoses, respectively (241). Regions where flow separation or flow obstruction occurs result in increased viscous energy loss, as seen in FIGURE 30 (241). To put these energy losses in perspective, the hydraulic work done by the left ventricle is on the order of 1.6 W (120 mmHg × 6 L/min): ∼1.6 W is needed to pump 6 L/min at 120 mmHg (242). To put it another way, the heart could compensate for 10 mW of energy dissipation by increasing the mean pressure by <1 mmHg.

### 5.3. Fluid and Solid Mechanical Analysis

#### 5.3.1. Experimental methods.

**FIGURE 31.**
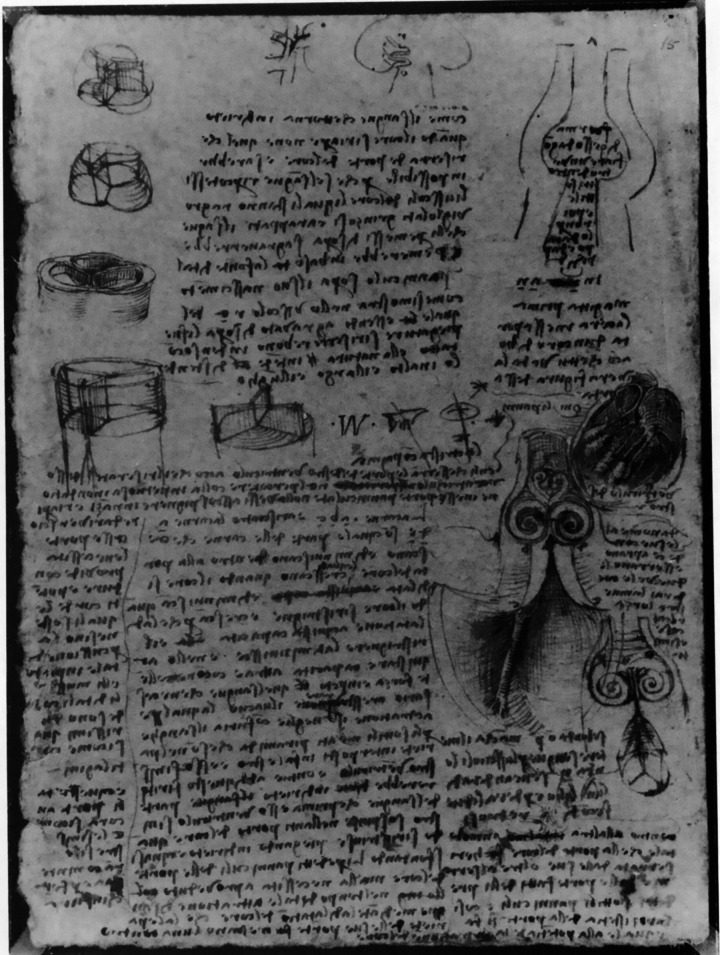
Leonardo da Vinci’s sketches of the aortic valve (*left*) and the vortices in the sinuses that push the leaflets together (*right*). Adapted from Ref. 243, with permission from *The Annals of Thoracic Surgery*.

There are also several experimental methods applied to quantifying the structural mechanics and hemodynamics of the aortic valve. Identifying any changes in the valve dynamics or alteration of flow patterns would indicate abnormal valve function. Qualitative visualization of the aortic valve and physiological flows has been practiced for a long time, dating back to the fifteenth century, when Leonardo da Vinci first drew vortices in the aortic root (FIGURE 31) (243).

More recent in vitro methods include laser Doppler velocimetry. However, it was only capable of capturing pointwise information. The introduction of digital particle image velocimetry (PIV), which increased the time resolution, substantially improved the understanding of flow around heart valves and the guidance of prosthetic valve development (244). The PIV techniques were greatly enhanced by the introduction of the multiplanar PIV, which made possible the measurement of flow in three dimensions. These techniques are reviewed in detail by Kaminsky et al. (245). To study correlation between calcification and shear stress, and more specifically the speculation of a higher chance of calcification because of the lower shear stresses due to the lack of coronary flow on the noncoronary leaflet, Yap et al. (218, 246) used a combination of laser doppler velocimetry (LDV) and an in vitro pulsatile flow loop to quantify fluid shear stresses affecting the leaflets. The valve model was mounted in a flow loop made of clear acrylic. The LDV system was then used to estimate shear stresses based on velocities measured at different locations throughout the cardiac cycle. Shear stresses on the aortic side of the leaflets were shown to be higher in systole than in diastole. Systolic shear stress increased with a higher stroke volume or a wider valve opening and decreased with a higher heart rate. The shear stresses on the ventricular side exhibited reversal of direction in late systole (218, 246).

**FIGURE 32.**
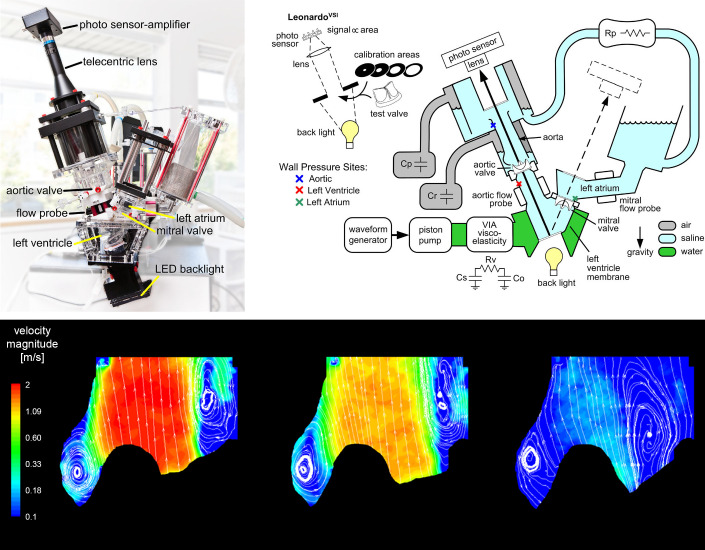
*Top*: in vitro hydrodynamic tester (ViVitro Pulse Duplicator system; ViVitro Labs, Inc., Victoria, BC, Canada) used to study the biomechanics of different cardiac valves. Adapted from Ref. 247, with permission per Open Access terms. *Bottom*: velocity contours and particle paths determined in vitro by laser particle image velocimetry (PIV). Adapted from Ref. 176, with permission per Open Access terms.

Hydrodynamic pulse duplicator systems are also used to assess valve kinematics and hemodynamics by reproducing the physiological pressures and pulsatile flow through the valves. These systems can be further used to support and validate the results of computational simulations when both their setup and results are in reasonable agreement. Griffith et al. (247) used the ViVitro Pulse Duplicator system (ViVitro Labs, Inc., Victoria, BC, Canada), seen in FIGURE 32, along with high-speed videography to assess valve kinematics. Projected dynamic valve area (PDVA) data were then reconstructed via automatic image analysis and compared with those produced by their FSI simulation. Such an apparatus also provides measurements of flow rates and pressures across the valve over the cardiac cycle (247).

#### 5.3.2. Computational biomechanics.

Advances in computing hardware and software have facilitated the development of computational codes capable of simulating AVA dynamics. Codes for simulating fluid flow were initially developed separately from those that simulated structural deformations. The AVA provides a good example of a situation in which the flow patterns both depend on and affect the structural mechanics, and vice versa. The use of coupled FSI computational capabilities, beginning in the early 2000s (248), has been adapted for both research and prosthetic device development. However, given the complexities and challenges presented by the AVA structures in health and disease, FSI simulations require careful, skilled application to ensure context-specific relevance. Ensuring relevance requires the two-pronged approach of verification and validation (249, 250).

Verification refers to the assessment that the computational algorithms used to derive solutions to the governing equations have provided mathematically accurate results to within a specified tolerance. There are internationally accepted standards for this process within the ISO 9001 hierarchy, under which many commercially available codes have been developed. Verification might be done by comparison to exact solutions or previously verified codes. Comparison to experimental data, typically from in vitro models because of the ability to control the flow conditions and known material properties, can be useful provided the uncertainties in the experimental data have been appropriately quantified.

Validation refers to the process of assessing the degree to which verified results are applicable to addressing the originally stated purpose. Do the results provide a useful answer to a physiological or pathological question? Do they support (or refute) a hypothesis? These are context-specific judgments that depend on how the answers to these questions are to be applied and are useful for informing, e.g., the tolerance specified in the verification process. Answers to research questions or hypotheses might be validated quite easily, say with parameter sensitivity analysis. If the results are to be used as part of a regulatory application or to inform medical treatment, the validation process should be rigorously executed and documented, including limitations in the applicability of the data. As with verification, internationally accepted standards exist for these processes, which apply to both experimental and computational studies. The Food and Drug Administration (FDA) first issued guidelines for inclusion of computational simulations in regulatory submissions in the late 1990s (251). McVeigh et al. (252) and Santiago et al. (253) describe how these processes can be applied to prosthetic heart valve design.

Properly performed FSI simulations can provide in-depth flow patterns and relevant quantities such as wall shear stresses (48, 217), estimates of mechanical stresses and strains that provide insight on structural mechanics (48, 254) and predict changes in function based on altering input parameters (255), which may be useful for preoperative planning (256). If executed correctly, computational models can provide useful insight into the effects of normal physiological variations in AVA properties via the ability to run multiple simulations with varying inputs (material properties and boundary conditions). Predictions of the consequences of pathological conditions and their treatments can provide useful guidelines for future research and development efforts. In both cases, the equivalent range of in vitro experiments or in vivo observations might take years to accumulate. Computational modeling has been used for studying the function of the aortic valve for >40 years. Several reviews on computational modeling of the cardiovascular system and cardiac valves have been published (48, 256–258). Most of the computational work done on aortic valves focuses on the leaflets’ geometry and material properties and their effect on function (48, 255, 259). This includes several studies on bioprosthetic valves (260–267) and BAV (168, 268).

An important consideration is the choice of using “average” models representative of a patient population or patient-specific geometries, a choice that is guided by the questions being posed (48, 158, 217, 250, 254–256, 269). Applying patient-specific vessel geometries and boundary conditions limits the results to that specific patient. Using data from normal human subjects would enhance the applicability of the study’s results to a wide range of patients, as in population studies. The choice of boundary conditions is governed not only by the availability of in vivo data (e.g., imaging) but also by the goals of the investigation (262, 270–273).

**FIGURE 33.**
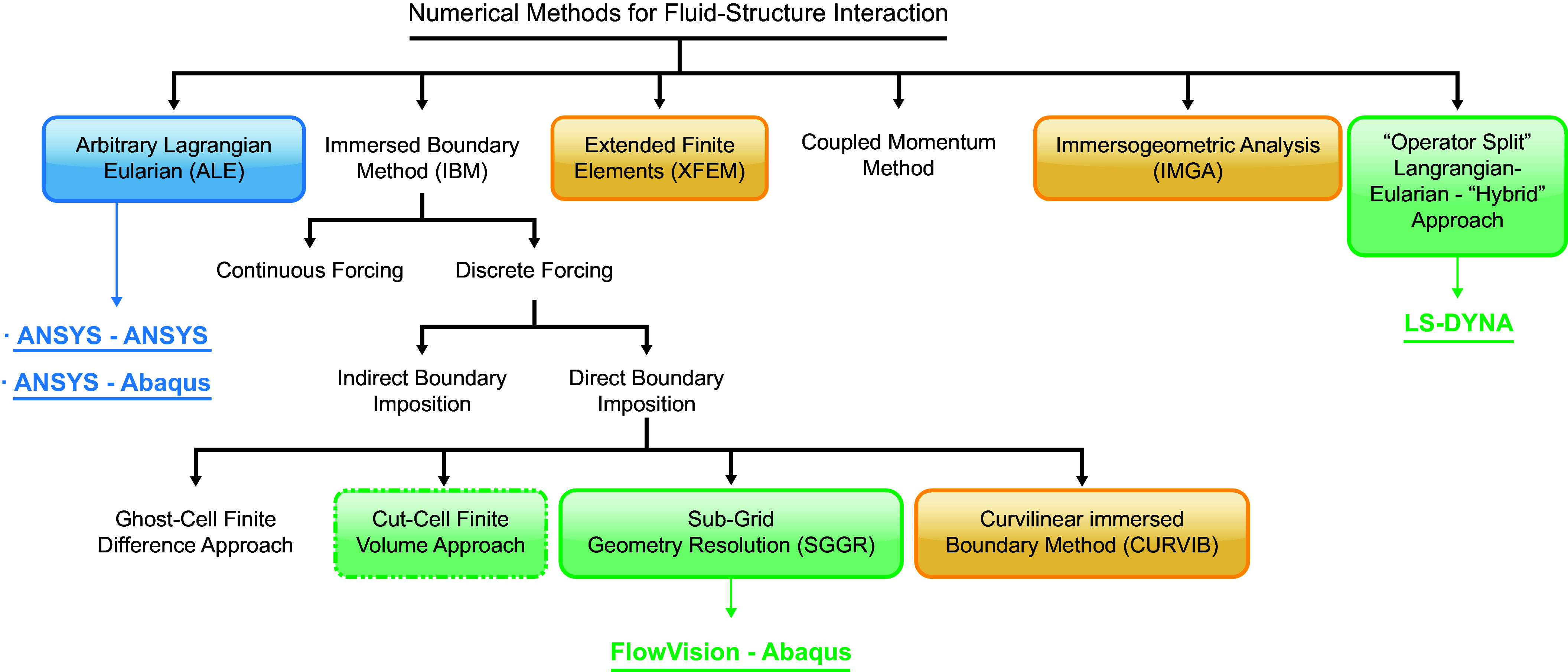
Flow chart of the different numerical methods for fluid-structure interaction (FSI). The arbitrary Lagrangian-Eulerian (ALE) method and its commercially available software are labeled in blue. Alternative methods that have commercially available software are labeled in green, and those that do not have commercially available software but are also used by the community are labeled in yellow.

The most widely used approach for heart valve FSI is the arbitrary Lagrangian-Eulerian (ALE) method such as that used in the commercial software ANSYS (Ansys, Inc., Canonsburg, PA). However, ALE comes with two main potential issues. First, the remeshing that occurs because of large deformations could create negative cell volume elements (48, 274). Second, the simulation might fail because of the split of the fluid domain during valve closure (48, 274). These can be overcome at a higher computational cost or by modifying the Navier–Stokes equations (48). Alternatively, other methods are used by commercially available software to overcome this problem. These include the immersed boundary (IB) method, used by FlowVision (Capvidia, Leuven, Belgium) when coupled with Abaqus (Dassault Systèmes SE, Vélizy-Villacoublay, France), and the “operator-split” Lagrangian-Eulerian approach, used by LS-DYNA (Ansys, Inc.) (48). The different types of numerical methods are illustrated in FIGURE 33.

## 6. CLINICAL RELEVANCE AND INTERVENTIONS

Preserving the native, normal, and healthy AVA anatomy and physiology is not just important to achieve healthy function but is also crucial to maintain the quality of life at both rest and exercise. To achieve this in disease, the different aspects of pathologies that affect the AVA should be studied and understood in order to correctly treat or intervene. Some of these pathologies are discussed in this section, while highlighting the use and impact of the different tools used to study the AVA. Recommended treatments and interventions are also discussed.

### 6.1. Bicuspid Aortic Valve

#### 6.1.1. Developmental biology.

Bicuspid aortic valve (BAV) is a genetic abnormality that can be detected during the early weeks of embryogenesis and is present at birth (275, 276). BAV could be associated with aortic root dilation and aortic coarctation and can increase the chance of calcification and valve degeneration as well as aortic dissection (277). The different morphological types of BAV (278) can influence prognosis and management (279, 280).

Many molecules are involved in the morphogenesis of the valve, such as *NOTCH-1* and endothelium-derived nitric oxide synthesis (eNOS), and there have been several studies trying to understand why the bicuspid valve is a heritable disease both clinically and experimentally (281, 282). *NOTCH-1* is a signaling molecule on the cell membrane, which when activated has the ability to affect gene expression in the nucleus. Mutations in *NOTCH-1* have shown to cause BAV, calcification, or aneurysm of the aorta (282). However, these mutations are only evident in ∼5–7% of BAV patients. and therefore they are not the only cause of BAV (146). eNOS is also another important molecule required for proper tricuspid valve formation. eNOS protein expression in aortic endothelial cells of patients with BAV has been shown to be much lower than in those with tricuspid aortic valves (114). There are several molecules involved in the development of BAV, and any alterations or reductions of these molecules can produce different morphologies of the valve (146).

#### 6.1.2. Flow dynamics.

**FIGURE 34.**
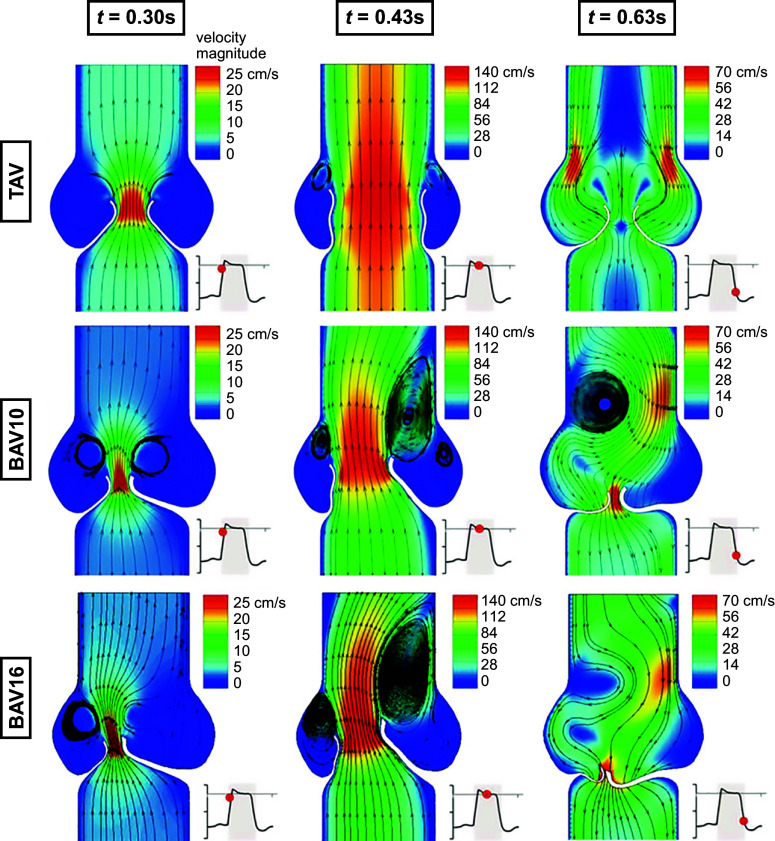
Fluid-structure interaction (FSI) simulations showing the different flow patterns through the aortic valve apparatus (AVA) for 2-dimensional (2-D) representations of a tricuspid valve (TAV), type 1 bicuspid aortic valve (BAV) with 10% eccentricity (BAV10), and type 1 BAV with 16% eccentricity (BAV16). Adapted from Ref. 213, with permission from *Biomechanics and Modeling in Mechanobiology*.

The left ventricle, the aortic valve apparatus, and the ascending aorta constitute a closely linked dynamic system that performs sophisticated functions that are highly centered in the aortic root, dependent on its complex physical characteristics that involve blood flow. Blood flow can be studied with tools provided by modern imaging techniques, such as 4-D flow MRI, and computational modeling, such as FSI and CFD, that can provide insights such as wall shear stress (283). FSI and CFD are extremely useful in defining the pattern of flow in different morphological types of BAV (FIGURE 34) (213).

**FIGURE 35.**
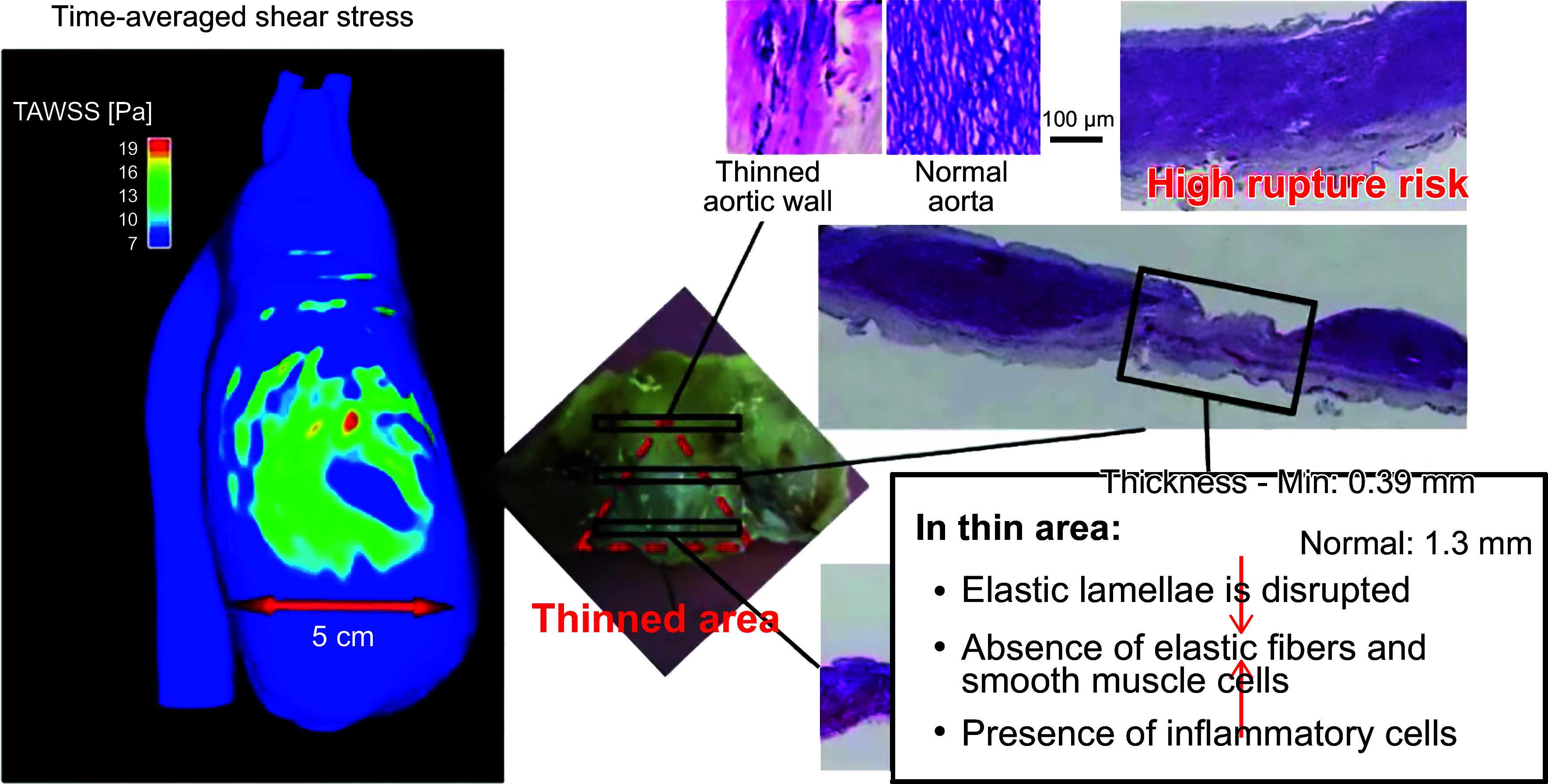
Right and noncoronary (R-N) cusp fusion in bicuspid aortic valve (BAV). *Left*: computational fluid dynamics (CFD) simulation showing time-averaged wall shear stress (TAWSS) for a BAV case with aneurysm and an aortic jet aimed at the aortic wall at a very high velocity (>3 m/s), causing high shear stress on the wall, which could be very damaging to the wall. *Right*: image and histology of the thinned area for the tissue sample collected from the same patient at the flow impingement on the wall, with a minimum thickness of 0.39 mm as opposed to a normal thickness of 1.3 mm. Adapted from Ref. 283, with permission per Open Access terms.

When the gap between the open leaflets is narrower, the flow must squeeze more to get through. This could form a strong turbulent jet that might impact the aortic wall, leading to a potential weakening factor due to the high shear stress on the wall. This weakening factor can cause aortic aneurysm or dissection due to thinning of the wall or loss of the medial layer at the impingement of the jet on the wall, as shown in FIGURE 35. Right and noncoronary (R-N) cusp fusion can cause aortic aneurysm due to the high velocity of the blood coming through the valve going straight to the aortic wall, which causes high shear stress (FIGURE 35). In contrast, right and left coronary (R-L) cusp fusion results in high-velocity flow going through the middle of the valve and aorta, without impacting the wall, and hence the wall shear stresses are not very high (283). BAV cusp morphology has a major impact on the pattern of blood flow and WSS in the ascending aorta (284, 285).

**FIGURE 36.**
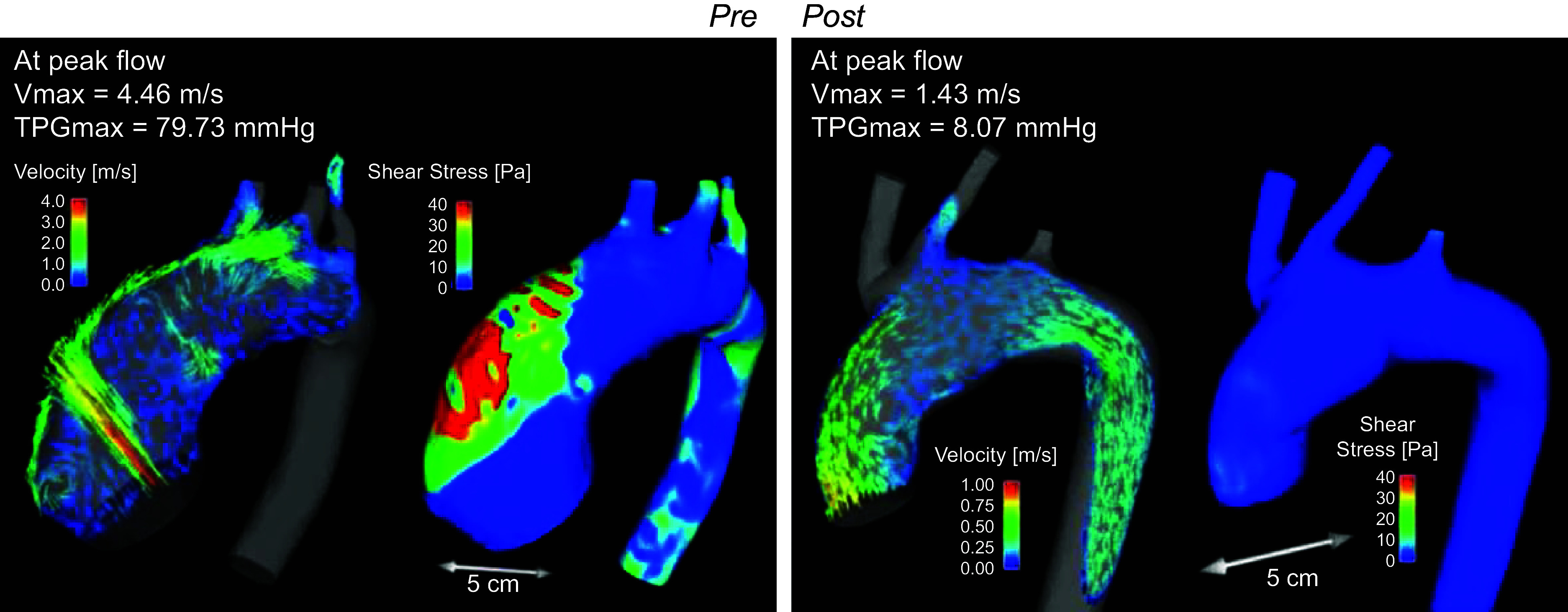
Pre- and postoperative computational fluid dynamics (CFD) simulations for a right and noncoronary (R-N) cusp fusion and aortic aneurysm patient (treated with a freestyle porcine xenograft root and a Dacron graft) showing the blood flow velocity and wall shear stresses at peak flow. TVP, transvalvular pressure gradient. Adapted from Ref. 283, with permission per Open Access terms.

This R-N cusp fusion and ascending aortic aneurysm case was surgically treated with a freestyle porcine xenograft root and a Dacron graft to replace the diseased valve and ascending aorta, respectively, which resulted in smoother flow patterns that follow the aorta with lower shear stresses and without the strong aortic jet impacting the wall evident preoperatively (FIGURE 36) (283).

#### 6.1.3. Treatments.

**FIGURE 37.**
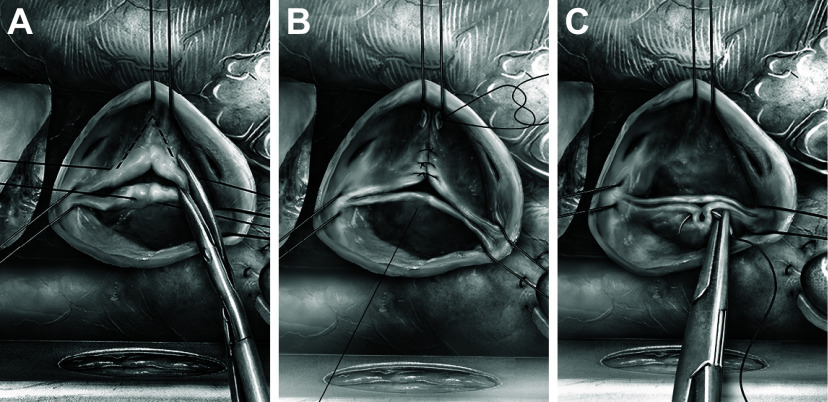
Different methods of repair techniques for bicuspid aortic valve (BAV). *A*: wedged shaped excision of fused cusp. *B*: plication of sinus base with pledgeted suture. *C*: plication of noncoronary cusp. Adapted from Ref. 286, with permission from *Operative Techniques in Thoracic and Cardiovascular Surgery*.

Treatments to substitute the BAV include prosthetic (such as mechanical valves), bioprosthetic [such as porcine valves and transcatheter aortic valve implantations (TAVI)], homograft or autograft (such as the Ross procedure) valves (283). BAV is more commonly treated with valve replacement; however, it can be effectively repaired (286–288) (FIGURE 37).

### 6.2. Degenerative Aortic Valve Disease

Degenerative aortic valve disease (DAVD) is a common disease in the elderly, especially in the Western world. Prevalence of DAVD characteristics, such as aortic valve sclerosis, calcification, thickening, or stenosis, in people above 65 yr of age was reported to be between 21% and 31%. Progressive obstruction of the LVOT often complicates DAVD and may lead to LV pressure overload, congestive heart failure, syncope, and sudden death. DAVD is a progressive disease that starts with sclerotic degenerations of the valve caused by dynamic changes of the valvular tissue (289, 290).

Early lesion of aortic valve calcification involves basement membrane disruption, similar to atherogenesis or atherosclerotic disease. Endothelial dysfunction is suggested to play a role in the calcification that is predominantly on the aortic side of the valve. The flow on the aortic side of the valve is turbulent, suggesting that the interaction between the shear stress and the endothelial cells there contributes to calcification. Genetic factors also have a role in the risk of DAVD and provide a link to the pathology of valve calcification. As mentioned above, gene mutations in NOTCH1 transcriptional factor may lead to calcification and have been found in patients with severe valvular calcification. It has also been found that shorter leukocyte telomere length is associated with calcification of the aortic valve (290).

New imaging modalities, such as contrast-enhanced molecular imaging techniques used as an adjunct for cardiac magnetic resonance imaging (CMR), may better define the pathological pathways involved in DAVD. Lipid-based gadolinium-complexed nanoparticles can be used to enhance CMR’s detection and characterization of atheromatous plaque. Micro-CT can also be used for ex vivo imaging and assessment of mineral levels of dysfunctional valves, such as in calcification (290).

There are several therapeutic interventions to delay or prevent DAVD’s progression. These include risk factor modification, the use of HMG-CoA reductase inhibitors (statin therapy), the use of angiotensin-converting enzyme (ACE) inhibitors, and the use of matrix metalloproteinase (MMP) inhibitors. Interventional techniques are commonly used at the end point of DAVD management, which usually involve surgical repair or valve replacement using mechanical or bioprosthetic valves. Other interventions include tissue engineering, percutaneous valve replacement, and stem cells (290).

### 6.3. Rheumatic Aortic Valve Disease

**FIGURE 38.**
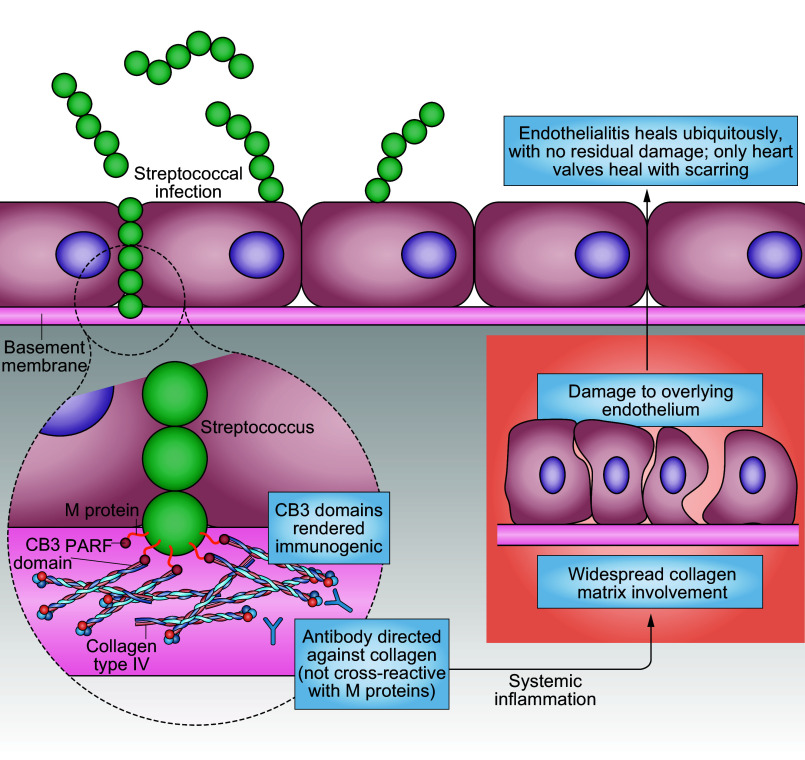
Proposed pathogenesis of rheumatic fever. Adapted from Ref. 291, with permission from *Nature Reviews Cardiology*.

Rheumatic fever causes a specific type of pathophysiology caused by an abnormal protein (291). Rheumatic heart valve disease (RHVD) affects the aortic valve and is a postinfectious sequel of acute rheumatic fever resulting from an abnormal immune response to a streptococcal pharyngitis that triggers chronic valvular damage. It predominantly affects children and young adults, mainly women in underdeveloped and developing countries (FIGURE 38). Rheumatic aortic valve disease causes inflammation in the leaflets that results in fibrosis, thickening, and shrinkage of the cusps, as well as sometimes commissural fusion. This anatomic disruption of the AVA leads to valve dysfunction. Treatments of this disease usually involve valve replacement or repair (292–296).

### 6.4. Aortic Valve Prolapse

**FIGURE 39.**
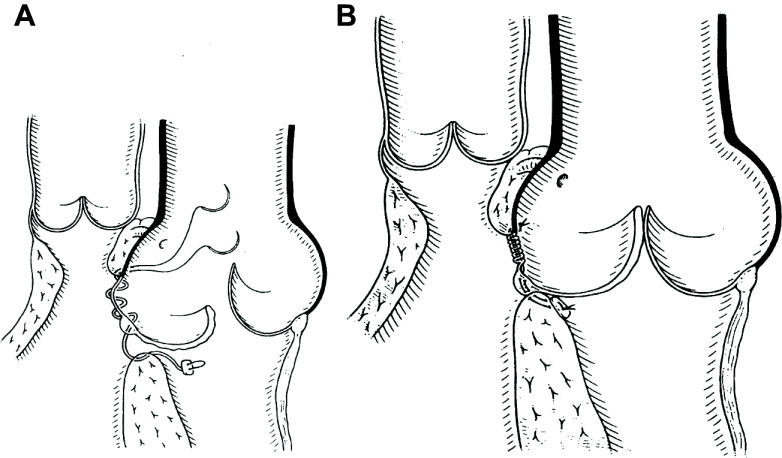
Anatomic correction of the syndrome of prolapsing right coronary aortic cusp, dilation of the sinus of Valsalva, and ventricular septal defect (VSD). Diagrams showing before repair (*A*) and after repair (*B*). Adapted from Ref. 92, with permission from *Annals of Thoracic Surgery*.

Aortic valve prolapse is “defined as downward displacement of leaflet material below a line joining the points of attachment of the aortic valve leaflets” (297). It usually causes aortic regurgitation, which is commonly associated with ascending aortic dilation. Prolapse of the right coronary cusp can be a component part of the syndrome of dilated right coronary sinus of Valsalva and ventricle septal defect (FIGURE 39) (92). Ventricle septal defect (VSD) is a defect found at birth of a hole in the septum between the two ventricles. Interventions to treat this prolapse usually involve valve repair or transcatheter VSD closure when the prolapse is due to a VSD (298–305).

### 6.5. Interventions

AVA’s fluid-structure interactions, structure-function relations, and dynamism and cross talk points discussed in this review are crucial to maintain normal and healthy valve function at both rest and exercise. Therefore, interventions to manage aortic valve diseases should aim to preserve full function by conserving, as much as possible, the morphology and mobility of the cardiovascular tissue across these wide-ranging conditions to maintain quality of life. A few of these surgical methods are mentioned below.

#### 6.5.1. Valve repair and valve-conserving operations.

Valve repair is an example of a surgical method that aims to preserve dynamism and cross talk. It consists of surgical techniques that aim to achieve homogeneous coaptation level for all leaflets by targeting normal size and shape of the leaflets and by fixing the mobility of the leaflets, while maintaining the native tissue and leaflets of the valve (287, 306, 307). Aortic valve repair for patients with aortic stenosis usually yields better left ventricle ejection fraction and lower 1-yr mortality than valve replacements. However, aortic valve repair usually has a higher reoperation rate at 1 yr (308).

Valve-conserving operations (VCOs) are surgical methods that preserve the native valve, and may use valve repair, for aortic aneurysms or dissection, which also aim at preserving dynamism and cross talk. These diseases usually affect the aortic wall of the sinuses and ascending aorta. In VCOs, the diseased aortic wall is normally replaced by a Dacron tube with near-native geometry and material properties, while preserving the aortic leaflets, such as in the remodeling Yacoub I and II operations and the reimplantation David procedure (269, 309).

#### 6.5.2. Fixed right ventricular outflow obstruction.

Other interventions that preserve dynamism and cross talk include excision of the fibrous tissue and mobilization of the fibrous trigones in dynamic and fixed subaortic stenosis (310). This preserves the integrity of the LVOT without the need for destructive methods such as the modified Konno operation (311).

#### 6.5.3. Valve replacements/substitutes.

The choice of valve replacements to preserve the dynamism of the AVA could lead to better and more optimized results. There are several limitations associated with the performance of current valvular interventions. Stented bioprosthetic valves lead to a rigid annulus, and mechanical valves result in unnatural hemodynamic performance (214). However, prosthetic valves have the material insufficiency of being nondegradable, which leads to calcification, fatigue, and rupture in the long term. The leaflet is still nonliving, with the usual mechanical and biological valve limitations (312).

Most bioprosthetic valves are made with bovine pericardium or porcine valve, such as the Medtronic Freestyle aortic root bioprosthesis. Although this biological material is blood compatible and reduces thrombosis risk, they evoke an immune response to both the xenogeneic (most valves) or allogeneic (homografts). All biological valves are subject to structural degeneration and failure within the first 10–15 yr after insertion (313, 314). In addition, biological valves are glutaraldehyde cross-linked to reduce immunogenicity and improve longevity; however, this also leads to calcification due to the presence of free aldehyde group and cross linking alters the stiffness of the valve (315, 316).

The only biological valve substitute that guarantees long-term survival of the inserted valve is the autograft (Ross operation) (317). This translates into extremely important clinical end points including enhanced survival and quality of life (318–324). However, the clinical application of this operation has been extremely slow (320). This is due to the operation being perceived as complex and the reported variable incidence of dilation of the aortic root when the autograft is inserted as a free-standing root (325). Recent technical modifications aiming at standardizing the operation and preventing late dilation have resulted in increased application of the operation (283, 321, 326, 327).

**FIGURE 40.**
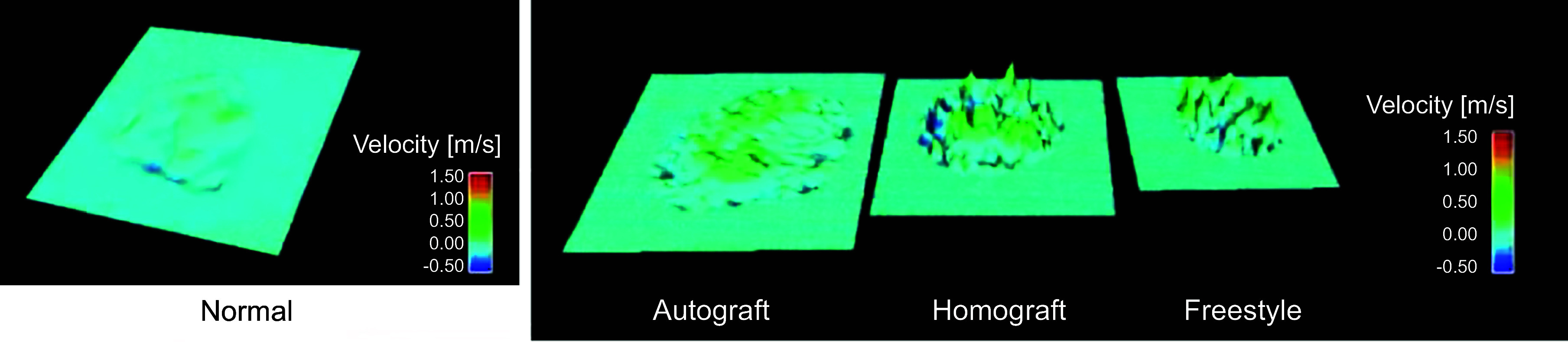
Velocity flow profiles for normal vs. intervention aortic flow mapping at the aortic valve; autograft profile is the most similar to the normal profile. Adapted from Ref. 283, with permission per Open Access terms.

The hemodynamic properties of the different valve replacements can be studied and compared by evaluating the blood flow through them. The representative velocity 3-D profiles through the open valve for normal cases shows a very smooth and flat hill shape bounded by the open valve orifice. For postoperative velocity profiles measured at the 10-yr follow-up, the autograft profiles have a flat and smooth shape, most similar to the normal profile, whereas the homograft and freestyle profiles show a sharper distribution with generally higher velocity values in the middle (FIGURE 40). The values for maximum flow velocity and orifice area ratio (*A*_orifice_/*A*_sinus_) of the autograft valves are also the closest to normal values, with the autograft valves having lower maximum flow velocities and larger orifice ratios than the other substitutes. Furthermore, the autograft valves have lower wall shear stress on the ascending aorta walls in comparison to the other substitutes, even though they are fairly similar in the descending aorta (283).

#### 6.5.4. Tissue-engineered heart valves.

As a solution to the drawbacks of biological bioprosthetic and mechanical valves and the nondegradable materials of the prosthetic valves, tissue-engineered heart valves (TEHVs) aim to produce regenerative heart valves with long durability, growth, adaptation, and ability to self-repair, without medical complications. Tissue-engineered valves aim to solve the problems faced by current valve replacements or substitutes and begin to function immediately upon implantation and continue to do so as they take on the properties of the native tissue over time (328–331). Furthermore, TEHVs aim to recapture the hemodynamic performance of the original living valve. Parker and Ingber (332) review the various heart tissue hierarchical structural networks that regulate the living cells and tissue, and microfabrication techniques to achieve them, as well as design principles that may be useful for heart tissue engineering.

**FIGURE 41.**
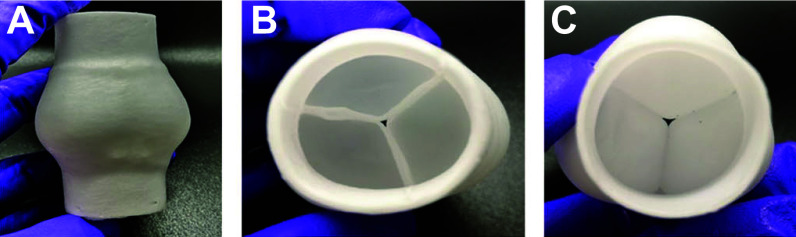
Side view (*A*), arterial view (*B*), and ventricular view (*C*) of the Magdi Yacoub Institute’s tissue-engineered heart valve (TEHV) named “Heart Biotech Composite Component Valve” (HCCV). Adapted from Ref. 346, with permission per Open Access terms.

Many TEHV attempts have used allogeneic or autologous cells and biological scaffolds, which come with practical and ethical drawbacks (333). In situ regeneration (334, 335) overcomes these problems faced by allogeneic or autologous tissue engineering by using scaffolds that attract endogenous cells after implantation and avoid the use of exogenous cells (330, 336–341) and is therefore the current approach in TEHV. At the Magdi Yacoub Institute, we are currently developing an in situ TEHV, named the “Heart Biotech Composite Component Valve (HCCV)” (342–345), and its manuscript is currently in preparation for publication (FIGURE 41) (346).

The advances in bioprinting and rapid prototyping techniques allow for dimension-specific valvular root structure, while utilizing the knowledge of dynamism of the aortic root (336). However, in vivo morphological and mechanical property changes of the implanted TEHV will have an effect on the performance of the construct, and therefore design considerations should take this into account (329, 347). The TEHV construct material choice will need to satisfy cellular recruitments while mimicking valvular material performance. Furthermore, after TEHV implantation, the hemodynamic and cellular interaction could lead to further changes in structure and function, even overriding the original structural design (336, 348). Design and computational modeling are used to attempt to offer predictions of these changes; however, it is still a challenging area, as rate and mechanism of spatial cellular matrix remodeling still remain elusive (349–351).

## 7. LIMITATIONS

The limitations of experimentational and computational approaches to understanding the complex physiological and pathological biomechanics depend on the reliability of the hardware and software and the skills of the users. Intermediate verification steps provide a rational assessment of the uncertainties in the resulting data. Validation that the results have addressed the clinical issues requires close collaboration between engineers and clinicians (250).

## 8. CONCLUSIONS

This article demonstrates the inherent elegance coupled with complexity of the AVA integrated functional unit. This could have important physiological implications for understanding the multiple sophisticated functions of the AVA, which translates into important clinical applications.

This review presents the application of engineering and basic science to clinical practice in our group, which includes Imperial College London, Aswan Heart Centre, and Magdi Yacoub Institute.

## GRANTS

Hussam El-Nashar is supported by a Ph.D. educational grant from Al Alfi Foundation, and Malak Sabry is also supported by a Ph.D. educational grant from Siemens Healthineers.

## DISCLOSURES

No conflicts of interest, financial or otherwise, are declared by the authors.

## AUTHOR CONTRIBUTIONS

H.E., M.S., and N.F. prepared figures; H.E., M.S., Y.-T.T., N.F., K.H.P., J.E.M., and M.H.Y. drafted manuscript; H.E., Y.-T.T., N.L., K.H.P., J.E.M., and M.H.Y. edited and revised manuscript; H.E., M.S., Y.-T.T., N.F., N.L., K.H.P., J.E.M., and M.H.Y. approved final version of manuscript.

## Footnotes

^1^
We adopt the definition provided in the Miller-Keane medical dictionary, wherein “apparatus” is “an arrangement of a number of parts acting together to perform a special function” (1).
